# Eighteen mitochondrial genomes of Syrphidae (Insecta: Diptera: Brachycera) with a phylogenetic analysis of Muscomorpha

**DOI:** 10.1371/journal.pone.0278032

**Published:** 2023-01-05

**Authors:** Hu Li, Yan Yan, Juan Li

**Affiliations:** Shaanxi Key Laboratory of Bio-Resources, State Key Laboratory of Biological Resources and Ecological Environment of Qinling-Bashan, School of Biological Science & Engineering, Shaanxi University of Technology, Qinling-Bashan Mountains Bioresources Comprehensive Development C.I.C., Hanzhong, Shaanxi, China; Sichuan University, CHINA

## Abstract

In this study, 18 mitochondrial genomes (mitogenomes) of Syrphidae were sequenced. These mitogenomes ranged from 15,648 to 16,405 bp and contained 37 genes that were similar to those from other Syrphidae species. Most protein-coding genes (PCGs) started with a standard ATN codon and ended with TAA/G. All transfer RNAs (tRNAs) could be folded into the cloverleaf secondary structure except *tRNA-Ser* (AGN), which lacks a dihydrouridine arm. The secondary structures of ribosomal RNAs (rRNAs) were predicted. Six domains (III is absent in arthropods) and 44 helices were included in the *16S rRNA*, and three domains and 24 helices were included in the *12S rRNA*. We found three conserved fragments in all syrphid mitogenomes. Phylogenetic analyses were performed based on the nucleotide data of 13 PCGs and two rRNAs from 76 Muscomorpha and three outgroup species. In results the paraphyly of Aschiza and Schizophora were supported, the Acalyptratae was also paraphyletic but the relationships of its superfamilies were difficult to determine, the monophyly of Calyptratea was supported with the relationships of Oestroidea and Muscoidea need to be further reconsidered. Within Syrphidae the monophyly of family level was supported, the Syrphinae were clustered into one branch, while the paraphyly of Eristalinae was still well supported.

## Introduction

The Muscomorpha (Diptera: Brachycera), an infraorder of Brachycera, consists of two groups, Aschiza and Schizophora, plus the unranked clade Cyclorrhapha [1–4]. The monophyly of several of these groups is debated. The evolution of Aschiza has been considered one of the most challenging phylogenetic questions in dipterology [1]. Aschiza contains two superfamiles: Syrphoidea and Platypezoidea. Syrphoidea was suggested to be paraphyletic in some previous studies [1,2,5], while Li [6], Pu *et al*., [7] and Skevington and Yeates [8] argued Syrphoidea is monophyletic. Schizophora underwent a recent, rapid radiation of lineages, and so represents most of the family-level diversity in Diptera. Relationships among Schizophora are poorly supported with the available data and their robust resolution remains a major challenge [2]. Schizophora is divided into two subsections, Acalyptratae and Calyptratea [4,7,9]. The Acalyptratae seemed paraphyletic in Ramakodi *et al*., [10] and Wiegmann *et al*., [2]. The monophyly of Calyptratea is supported, but most classifications of the Calyptratea divide the group into Hippoboscoidea, Muscoidea and Oestroidea, with the relationships between these superfamilies controversial [11]. In this study, we collected the published mitogenome data from each superfamily in Muscomorpha (Table 1), generated phylogenetic trees, and discussed their phylogenetic relationships to try to solve the current controversy.

**Table 1:** Information of the complete mitochondrial genomes used in this study.

Superfamily	Family	Species	Accession number	Size (bp)	Reference
Syrphoidea	Syrphidae	*Allograpta javana*	MZ286965	16,387	This study
		*Asarkina ericetorum*	MZ202394	16,080	This study
		*Betasyrphus serarius*	MZ202393	16,369	This study
		*Dideoides latus*	MZ315034	16,308	This study
		*Epistrophe bashanensis*	MZ420564	16,185	This study
		*Epistrophe lamellata*	MZ398236	16,405	This study
		*Epistrophe zibaiensis*	MZ361706	16,272	This study
		*Episyrphus balteatus*	KU351241	16,175	[7]
		*Eristalinus aeneus*	MH321208	16,245	[12]
		*Eristalinus barclayi*	MH321205	15,757	[12]
		*Eristalinus fuscicornis*	MH321204	15,815	[12]
		*Eristalinus quinquestriatus*	MT471322	15,872	NCBI
		*Eristalinus* sp.	MT942687	15,883	NCBI
		*Eristalinus tabanoides*	MH321207	15,792	[12]
		*Eristalinus vicarians*	MH321206	15,966	[12]
		*Eristalinus viridis*	MN494096	15,640	NCBI
		*Eristalis arbustorum*	MW172433	15,983	This study
		*Eristalis cerealis*	NC_050932	15,348	[13]
		*Eristalis himalayensis*	MW307783	16,008	This study
		*Eristalis tenax*	MH159199	15,996	[14]
		*Eupeodes corollae*	KU379658	15,326	[7]
		*Eupeodes confrater*	MZ272471	16,175	This study
		*Eupeodes latifasciatus*	MZ329813	15,968	This study
		*Eupeodes luniger*	MZ292750	16,182	This study
		*Ferdinandea cuprea*	MT834868	15,907	[15]
		*Helophilus virgatus*	MN148445	15,742	[16]
		*Korinchia angustiabdomena*	MK870078	16,473	[6]
		*Mallota bellus*	MW727270	15,763	This study
		*Mallota vilis*	MW727271	15,824	This study
		*Mallota viridiflavescentis*	MW172435	15,912	This study
		*Melanostoma orientale*	MN788095	16,229	[17]
		*Melanostoma scalare*	MT185683	16,380	[18]
		*Mesembrius niger*	MW690641	15,802	This study
		*Ocyptamus sativus*	KT272862	15,214	[19]
		*Orthonevra geniculata*	NC_050314	15,858	NCBI
		*Parhelophilus kurentzovi*	MW727269	15,648	This study
		*Phytomia errans*	MW172434	15,928	This study
		*Phytomia zonata*	MT478107	15,716	[20]
		*Platycheirus albimanus*	MT622646	16,648	[21]
		*Simosyrphus grandicornis*	DQ866050	16,141	[22]
		*Syrphus torvus*	MW074962	16,444	[23]
		*Syrphus vitripennis*	MT254536	16,185	[24]
		*Volucella nigricans*	MK870079	15,724	[6]
Syrphoidea	Pipunculidae	*Pipunculus* sp.	MF774216	14,619	NCBI
Platypezoidea	Lonchopteridae	*Lonchoptera multiset*	MF774215	14,614	NCBI
	Platypezidae	*Platypeza* sp.	MF774217	16,636	NCBI
	Phoridae	*Megaselia scalaris*	NC_023794	15,599	[25]
		*Apocephalus antennatus*	MG546669	18,674	[25]
Oestroidea	Oestridae	*Cephalopina titillator*	NC_046479	16,534	[26]
		*Rhinoestrus usbekistanicu*	NC_045882	16,534	[26]
	Tachinidae	*Elodia flavipalpis*	NC_018118	14,932	[27]
		*Subclytia rotundiventris*	NC_050880	15,574	[28]
	Calliphoridae	*Lucilia sericata*	AJ422212	15,945	[29]
		*Triceratopyga calliphoroides*	MK893471	16,529	[30]
	Sarcophagidae	*Sarcophaga impatiens*	NC_017605	15,169	[31]
		*Peckia collusor*	MH879763	15,233	[32]
Muscoidea	Anthomyiidae	*Fucellia costalis*	NC_042770	16,175	[33]
		*Hylemya vagans*	NC_050317	15,365	NCBI
	Muscidae	*Haematobia irritans*	NC_007102	16,078	NCBI
		*Musca domestica*	NC_024855	16,108	[34]
Sciomyzoidea	Sepsidae	*Nemopoda mamaevi*	NC_026866	15,878	[35]
	Sciomyzidae	*Trypetoptera punctulata*	MK644823	20,536	[36]
Lauxanioidea	Celyphidae	*Spaniocelyphus pilosus*	NC_034924	16,426	[37]
	Lauxaniidae	*Pachycerina decemlineata*	NC_034923	16,286	[37]
		*Cestrotus liui*	NC_034922	16,171	[37]
Tephritoidea	Tephritidae	*Bactrocera umbrosa*	KT881558	15,898	[38]
		*Bactrocera melastomatos*	KT881557	15,954	[38]
	Lonchaeidae	*Silba* sp.	MK913844	16,008	NCBI
		*Neoceratitis asiatica*	MF434829	15,481	[39]
Ephydroidea	Drosophilidae	*Drosophila koepferae*	MN551234	14,891	[40]
		*Drosophila montana*	HQ849820	15,137	NCBI
Opomyzoidea	Agromyzidae	*Liriomyza sativae*	NC_015926	15,551	[41]
		*Liriomyza trifolii*	GU327644	16,141	[42]
	Fergusoninidae	*Fergusonina taylori*	NC_016865	16,000	[43]
		*Fergusonina* sp.	HQ872008	16,000	NCBI
Hippoboscoidea	Hippoboscidae	*Melophagus ovinus*	NC_037368	15,573	[44]
Tabanoidea	Tabanidae	*Haematopota vexativa*	NC_059934	16,017	[45]
		*Atylotus miser*	NC_030000	15,858	[46]
	Rhagionidae	*Desmomyia sinensis*	NC_059816	16,430	[47]

Syrphidae, or hover flies or flower flies, is one of the larger families in Muscomorpha, and includes more than 6200 species around the world [48]. Most syrphid adults are flower visitors that feed on pollen and nectar, and many are important pollinators for natural ecosystems and agricultural crops [49,50]. Syrphid larvae, however, can be phytophagous, mycophagous, saprophagous, or predacious, and have diverse habitats [51]: phytophages can be found in the xylem of plants [52,53]; mycophages survive in fungal fruiting bodies [54]; saprophages live in environments rich in organic matter such as dung, nests of social Hymenoptera, decaying wood, and several types of freshwater bodies; and predacious syrphids are important natural enemies of aphids [55,56]. Several taxonomic classification systems for Syrphidae were built based on different morphological characteristics. The most well-known is the three-subfamily system comprised of Syrphinae, Eristalinae, and Microdontinae [1,53,57]. Some studies combining morphological and genomic data suggest the Syrphinae tribe Pipizini should be elevated to its own subfamily, Pipizinae [12,48,51,58]. Most previous studies agreed that Syrphinae, Pipizinae/Pipizini, and Microdontinae are monophyletic, while the monophyly of Eristalinae is debated [8,9,48,57].

The insect complete mitochondrial genome (or mitogenome) is a covalently closed, double-stranded DNA molecule consisting of 37 genes: 13 protein-coding genes (PCGs), 22 transfer RNAs (tRNAs), and two ribosomal RNAs (rRNAs) [59]. As they have some convenient characteristics, such as a small size (approximately 14–21 kb), lack of extensive recombination, conserved gene content, matrilineal inheritance, and relatively high evolutionary rate, complete mitogenomes are regularly applied to the study of comparative and evolutionary genomics and phylogenetic analysis [60–63].

In this study, we collected published mitogenome data from GenBank from every superfamily in Muscomorpha (Table 1). We then constructed phylogenetic trees to confirm the monophyly of Aschiza, Schizophora, and their inner superfamilies. We also added 18 new mitogenomes and collected all published mitogenomes of Syrphidae (ten Syrphinae and eight Eristalinae) to perform a more in-depth and comprehensive analysis of the phylogenetic relationship of Syrphidae.

## Materials and methods

### Sampling and DNA extraction

Samples of 18 species of Syrphidae (Table 1) that had been preserved in absolute ethanol and stored at -20°C were obtained from the preserved insect collections of the Museum of Zoology and Botany, Shaanxi University of Technology, Hanzhong, China. These were identified by Hu Li, Yan Yan, and Juan Li.

The specimens were dried after being removed from the absolute ethanol, and the legs and thoraxes of one specimen per species were used as samples for DNA extraction. The samples were manually ground into small particles (diameter ≤1 mm) using a grinding rod. Genomic DNA was extracted using a TIANamp Genomic DNA Kit (Tiangen, Beijing, China). The ground tissue was maintained at 56°C for six hours to completely lyse cells, while the remaining steps were conducted in accordance with the manufacturer’s protocol. The resulting genomic DNA was stored at −20°C.

### Sequencing assembly and annotation

The 18 complete mitogenomic sequences were sequenced using an Illumina NovaSeq 6000 platform and assembled with Geneious R9 [64]. Thirteen PCGs were identified using the invertebrate mitochondrial genetic code setting in ORF Finder, and PCG base composition and codon usage patterns were analyzed using MEGA 7 [65]. The locations and secondary structures of 22 tRNA genes were identified and predicted using tRNA scan-SE 1.21 [66] and ARWEN 1.2 [67]. Two rRNA genes were determined based on the locations of adjacent tRNAs genes and compared with the genes of other Syrphid mitogenomes, and the secondary structures of *16S rRNA* and *12S rRNA* were predicted according to data from other species [68–72]. Helical elements were predicted using ClustalX 1.81 and RNA Structure 5.2 [73]. The AT skew, GC skew, and A+T content were used to characterize the nucleotide composition of the mitogenomes [74,75]. Strand asymmetry was calculated using the following formulas: AT skew = (A–T) / (A+T), and GC skew = (G–C) / (G+C) [76,77].

### Phylogenetic analysis

To make a Muscomorpha mitogenome phylogenetic tree, we combined our 18 Syrphidae mitogenomes with all other complete, published Syrphidae mitogenomes, as well as two complete mitogenomes from each other family of Muscomorpha as representatives (Table 1) due to too many species in some families. Phylogenetic analyses of mitogenome data are known to be susceptible to compositional bias, particularly of the third codon positions in the PCGs [60], and particularly in the Diptera [22,31]. To reduce this bias, we built maximum likelihood (ML) and Bayesian inference (BI) phylogenetic trees based on three different datasets: (1) PCGs: all position of the PCGs (11607 bp); (2) PCG12: first and second codon positions of the PCGs (7582 bp); (3) PCG12RNA: the first and the second codon positions of the PCGs and two rRNA genes (9496 bp). The third codon was omitted from the latter two datasets because the third codon positions of PCGs may suffer from mutation saturation, which can lead to noise during phylogenetic analysis [78,79]. Because tRNA genes are highly conserved, they were not considered for phylogenetic tree construction.

Each of the PCGs was aligned with the MAFFT algorithm in Phylosuite [80] and translated according to the Invertebrate Mitochondrial genetic code, then the terminal codons were removed. The rRNA genes were aligned with MAFFT using the G-INS-i strategy [81], and the poorly aligned positions and divergent regions were removed using Gblocks 0.91b [82]. All sequences were assessed and manually concatenated using Phylosuite [80].

The heterogeneity of sequence divergence within the three datasets was analyzed using ALIGROOVE with the default sliding window size [83]. The best partitioning schemes and corresponding nucleotide substitution models were determined using PartitionFinder2 in Phylosuite [80] with the corrected Akaike information criterion (AICc) and greedy search algorithm [84].

Maximum likelihood (ML) analysis was performed using IQ-TREE [85], and evaluated using the ultrafast bootstrap approximation approach for 1,000 replicates. Bayesian inference (BI) analyses were performed using MrBayes 3.3.6 in Phylosuite, with two simultaneous Markov chain runs of 10,000,000 generations with sampling every 1000 generations. The phylogenetic trees were visualized using FigTree 1.4.2.

## Results

### Genome structure and nucleotide composition

**Fig 1:**
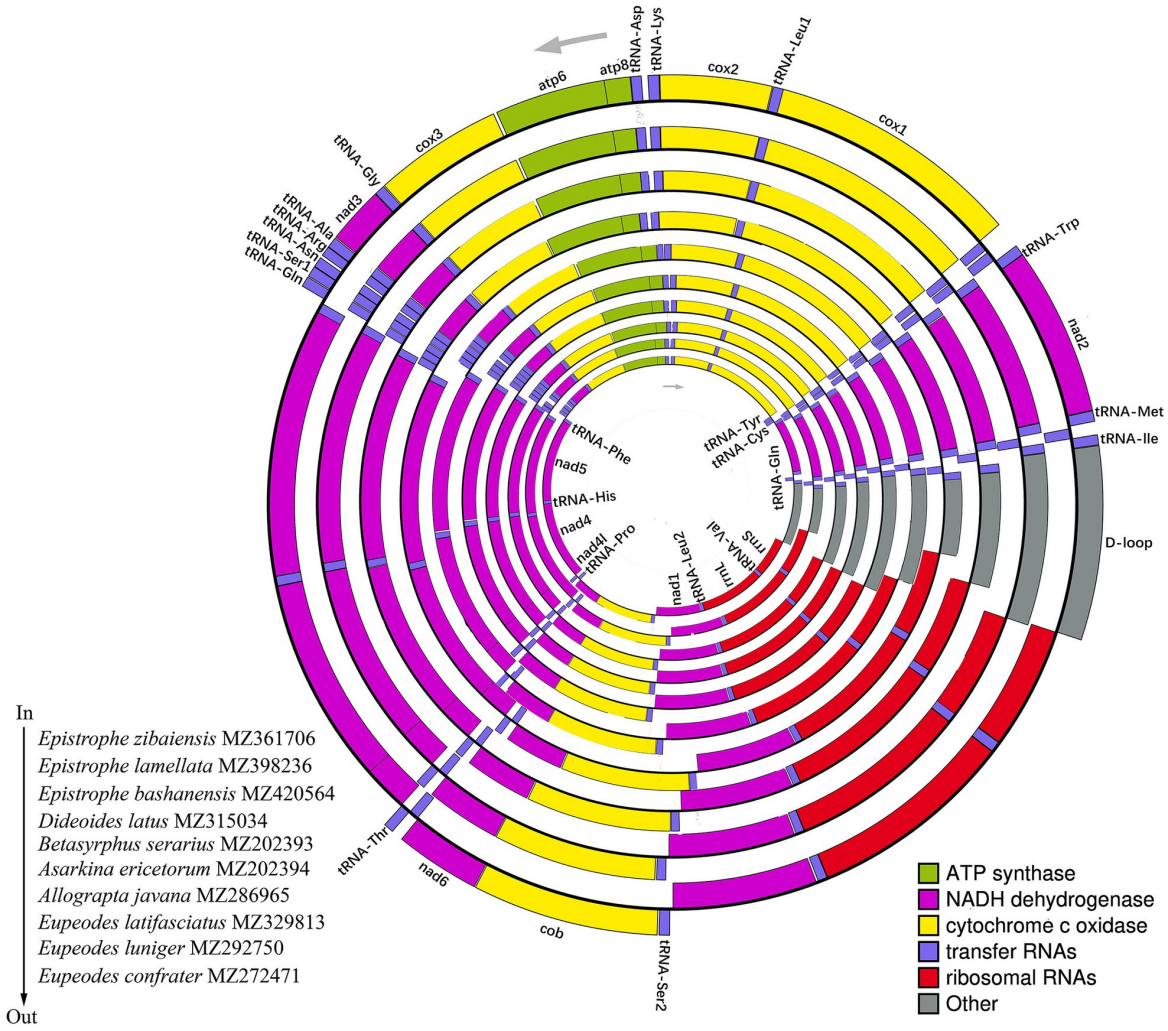
Circular map of the 10 Syrphinae species’ mitogenomes. The species from inside to outside are as follows: *Epistrophe zibaiensis* Huo, Ren et Zheng, 2007, *Epistrophe lamellata* Huo, Ren et Zheng, 2007, *Epistrophe bashanensis* Huo, Ren et Zheng, 2007, *Dideoides latus* Coquillett, 1898, *Betasyrphus serarius* Wiedemann, 1830, *Asarkina ericetorum* Fabricius, 1781, *Allograpta javana* Wiedemann, 1824, *Eupeodes latifasciatus* Macquart, 1829, *Eupeodes luniger* Meigen, 1822 and *Eupeodes confrater* Wiedemann, 1830.

**Fig 2:**
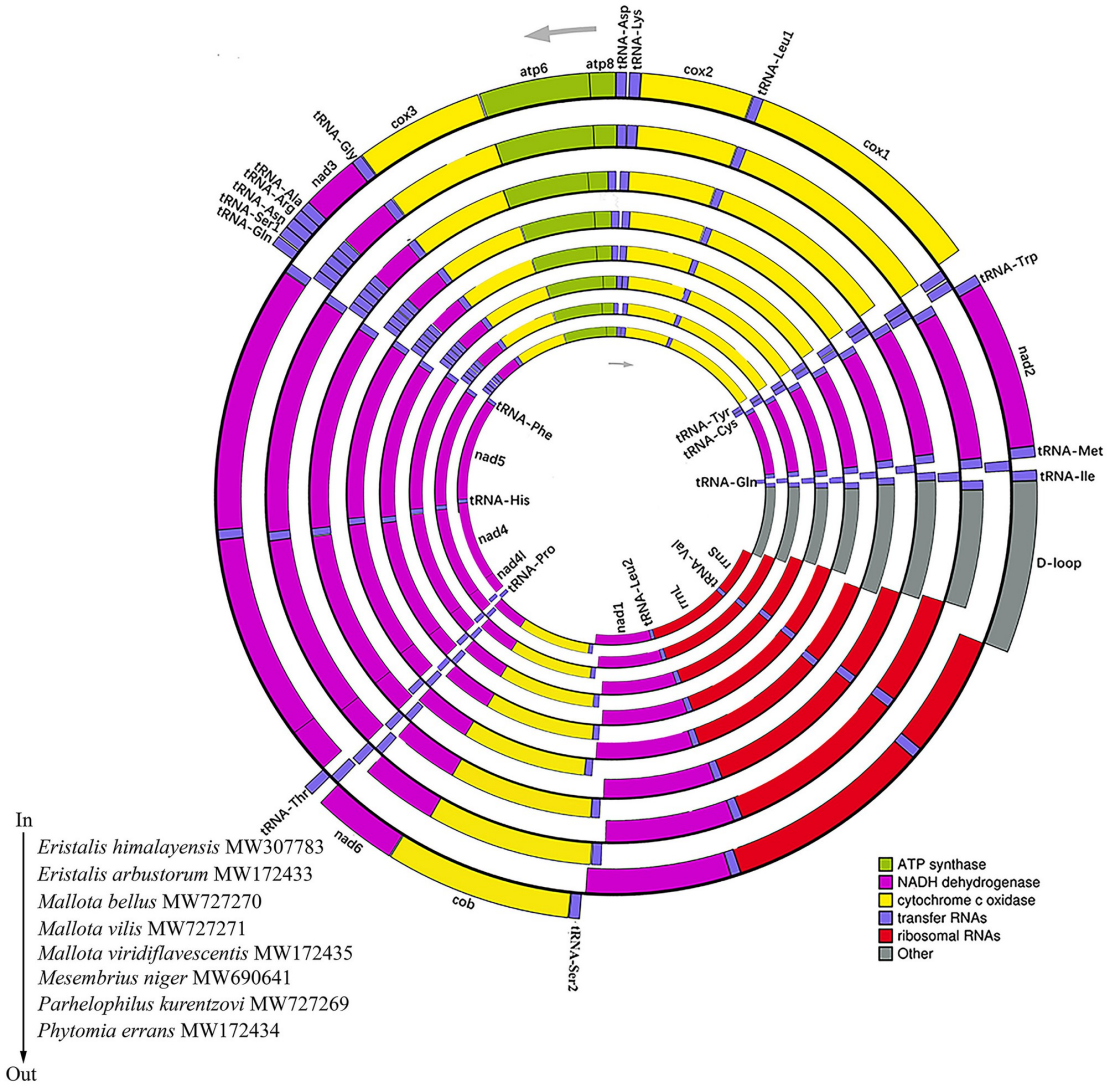
Circular map of the 8 Eristalinae species’ mitogenomes. The species from inside to outside are as follows: *Eristalis himalayensis* Brunetti 1908, *Eristalis arbustorum* Linnaeus, 1758, *Mallota bellus* Li, 1997, *Mallota vilis* Wiedemann, 1830, *Mallota viridiflavescentis* Huo et Ren, 2006, *Mesembrius niger* Sbiraki, 1968, *Parhelophilus kurentzovi* Violovitsh, 1960 and *Phytomia errans* Fabricius, 1787.

The 18 new mitogenome sequences have been deposited in GenBank (Accession Numbers in Table 1). Their gene order and organization were similar to previously published Syrphidae mitogenomes, containing 13 protein-coding genes (PCGs), 22 transfer RNAs (tRNAs), two ribosomal RNAs (rRNAs), and a D-loop region. A total of 23 genes (14 tRNAs and nine PCGs) are encoded on the majority strand (J-strand) and 14 genes (eight tRNAs, four PCGs, and two rRNAs) on the minority strand (N-strand) (S1–S18 Tables). The whole lengths of the 18 mitogenomes ranged from 15,648 bp (*Parhelophilus kurentzovi*) to 16,405 bp (*Epistrophe lamellata*) (Figs 1 and 2). The genes of *D-loop* that affect the difference in whole lengths (S1–S18 Tables). The mitogenome A+T contents ranged from 81.3% to 77.7%, and they displayed a strong A+T bias, which is typical of insect mitogenomes [59,64].

### Protein-coding genes

The A+T contents of the 13 PCGs combined for each species ranged from 75.3% to 79.1%. Most of the PCGs’ start codons used ATN (ATA/ATT/ATC/ATG), while a few PCGs were initiated by TCG, GTG, or TTG. Among the 18 species’ PCGs, *COX2*, *COX3*, *ATP6*, *ND4L* and *Cytb* used TAA as the stop codon, and all other genes ended with TAG or the incomplete forms TA- or T—(S1–S18 Tables).

**Fig 3:**
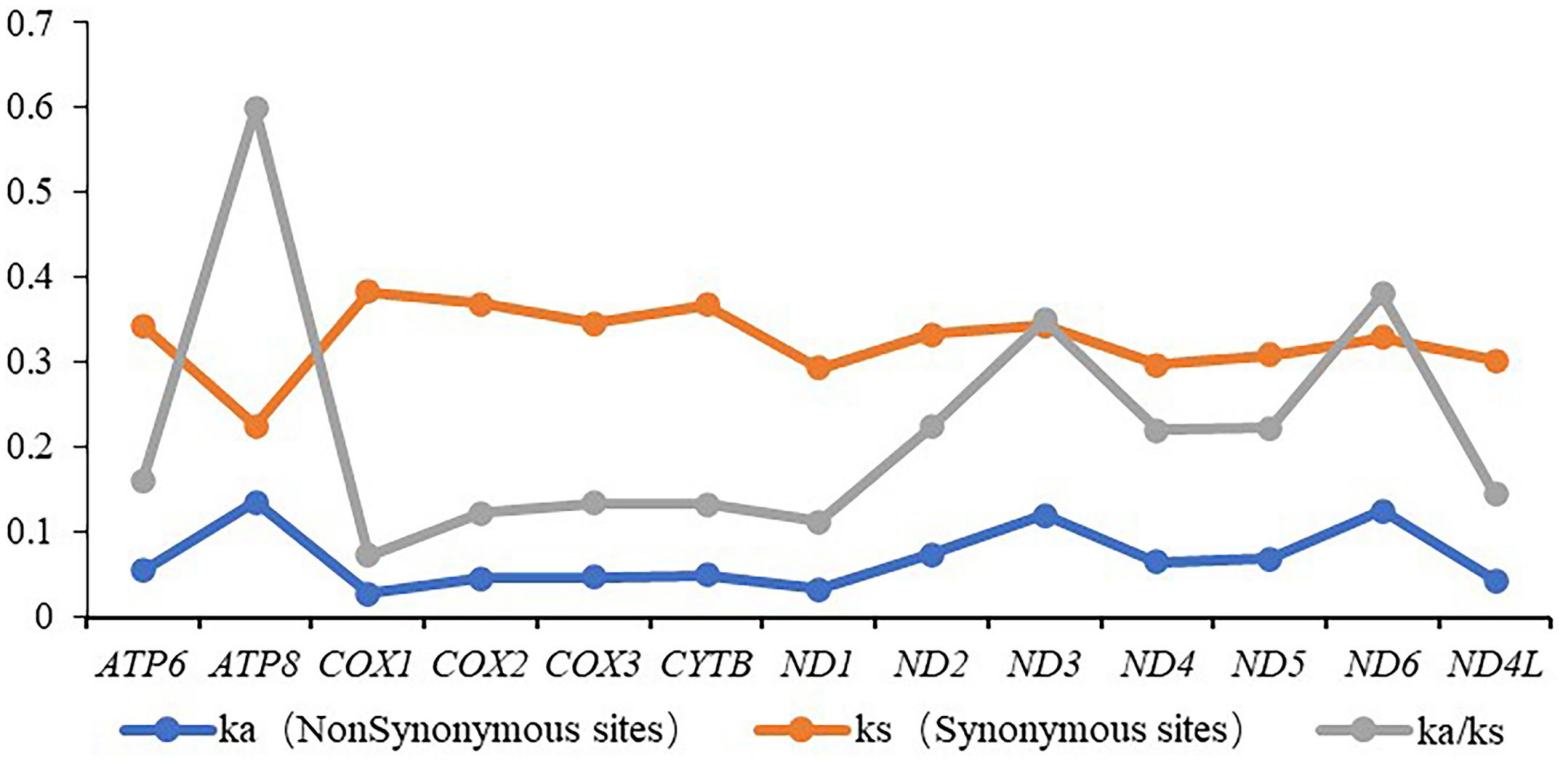
Evolutionary rates of 13 PCGs in the mitogenomes of the Syrphidae species.

In order to investigate the evolutionary rates of the 13 PCGs in Syrphidae, we calculated the value of Ka (non-synonymous substitutions), Ks (synonymous substitutions), and Ka/Ks for each PCG. The Ka/Ks value of all 13 PCGs were lower than 1, which indicated they are under relaxed selection pressure. The gene *ATP8* showed the highest evolutionary rate (Ka/Ks = 0.60), while *COXI* exhibited the lowest rate (Ka/Ks = 0.07) (Fig 3) as expected: for that reason, *COXI* has been used as a DNA barcode for the identification of various species [65].

### Transfer and ribosomal genes

**Fig 4:**
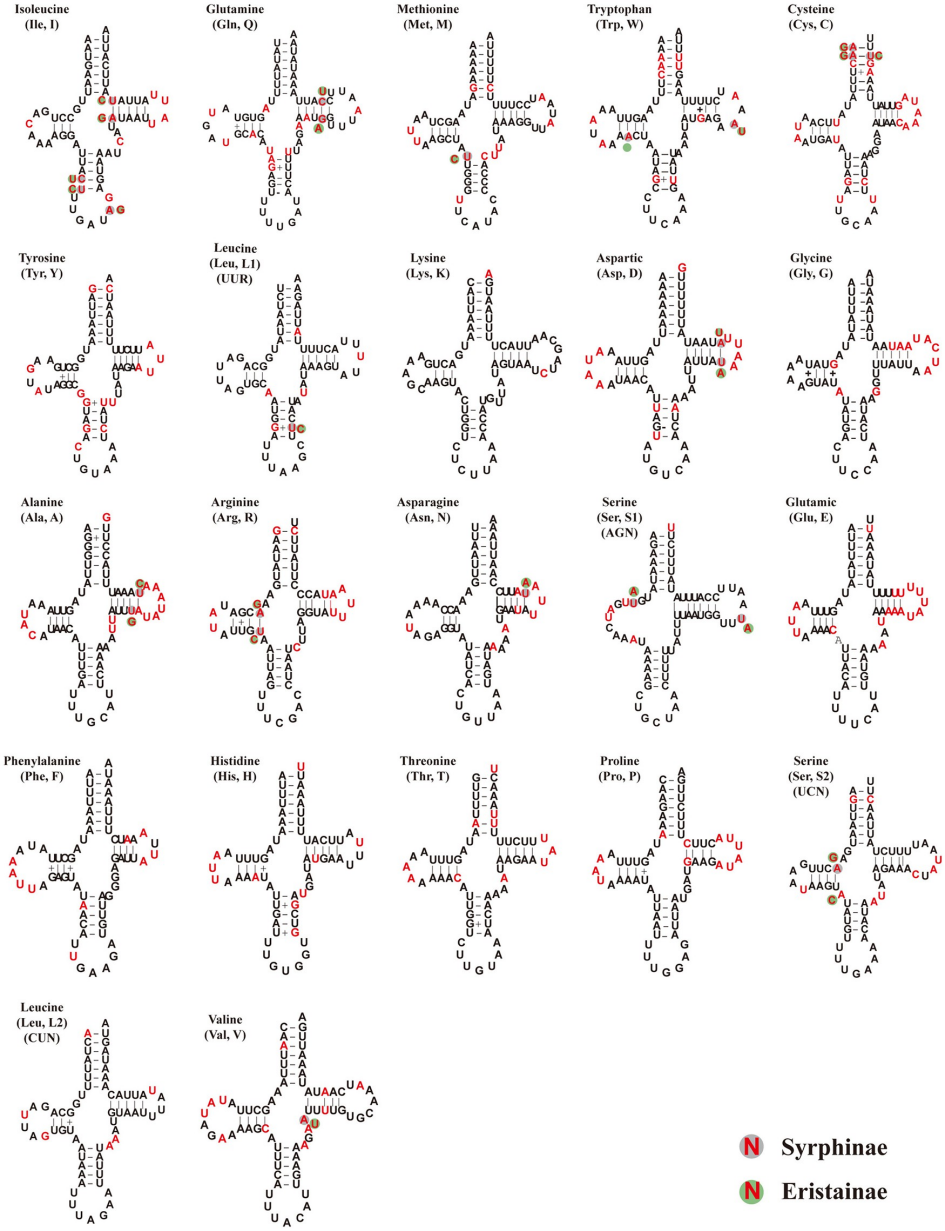
Predicted secondary structure of the 22 tRNAs in this study. The “-” indicates Watson-Crick base pairing and “•” indicates non-Watson-Crick base pairing. The conserved sites and variable sites are indicated with the black and red, respectively.

Each of the 18 syrphid species’ mitogenomes included 22 tRNAs: 14 were encoded on the J-strand and eight encoded on the N-strand. The lengths ranged from 64 to 80 bp. Their secondary structure suggests they can be folded into the typical clover-leaf structure of tRNAs, except for *tRNA-Ser1*, which lacked a dihydrouridine arm (DHU). Several unmatched base pairs (U-U, U-G, A-C, A-G, A-A) were also observed (Fig 4 and S1–S18 Figs). The lengths of each of the 22 types of tRNA were fixed across all eighteen species, and the sequences were also highly conserved. There were only individual site variations that were consistent across the subfamilies. For example: sites 27, 28, 36, 46 and 60 of *tRNA-Ile* were always C, U, A, A, U in Syrphinae, and U, C, G, G and C in Eristalinae. Other examples are shown in Fig 4. We suspected that the directed variation of these loci may correlate with the phylogeny of the species.

**Fig 5:**
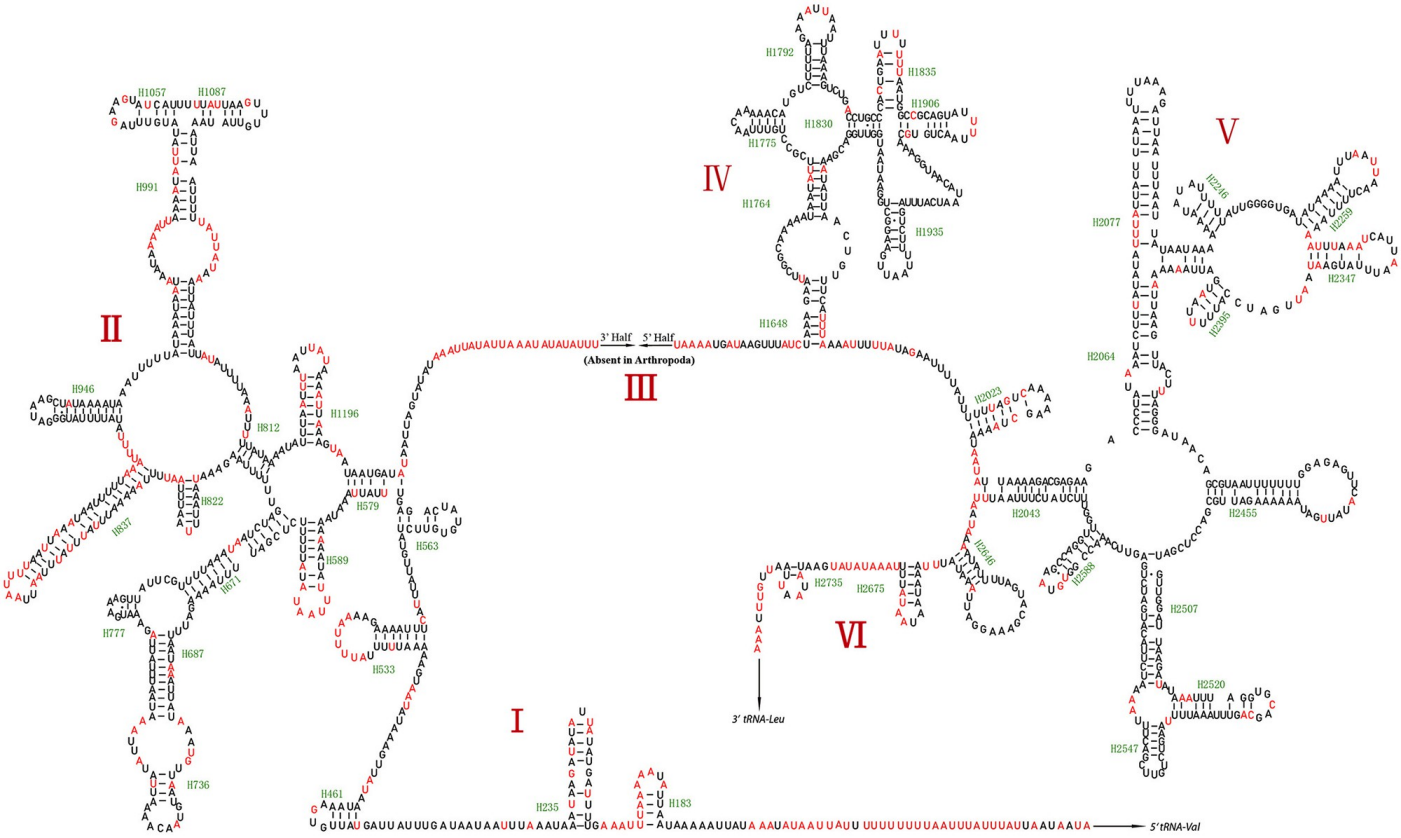
Predicted secondary structure of the 16S rRNA of the mitogenomes of this study. Dashes (-) indicate Watson-Crick base pairing, GU pairs are connected by dots (·), and the variable sites are indicated in red.

**Fig 6:**
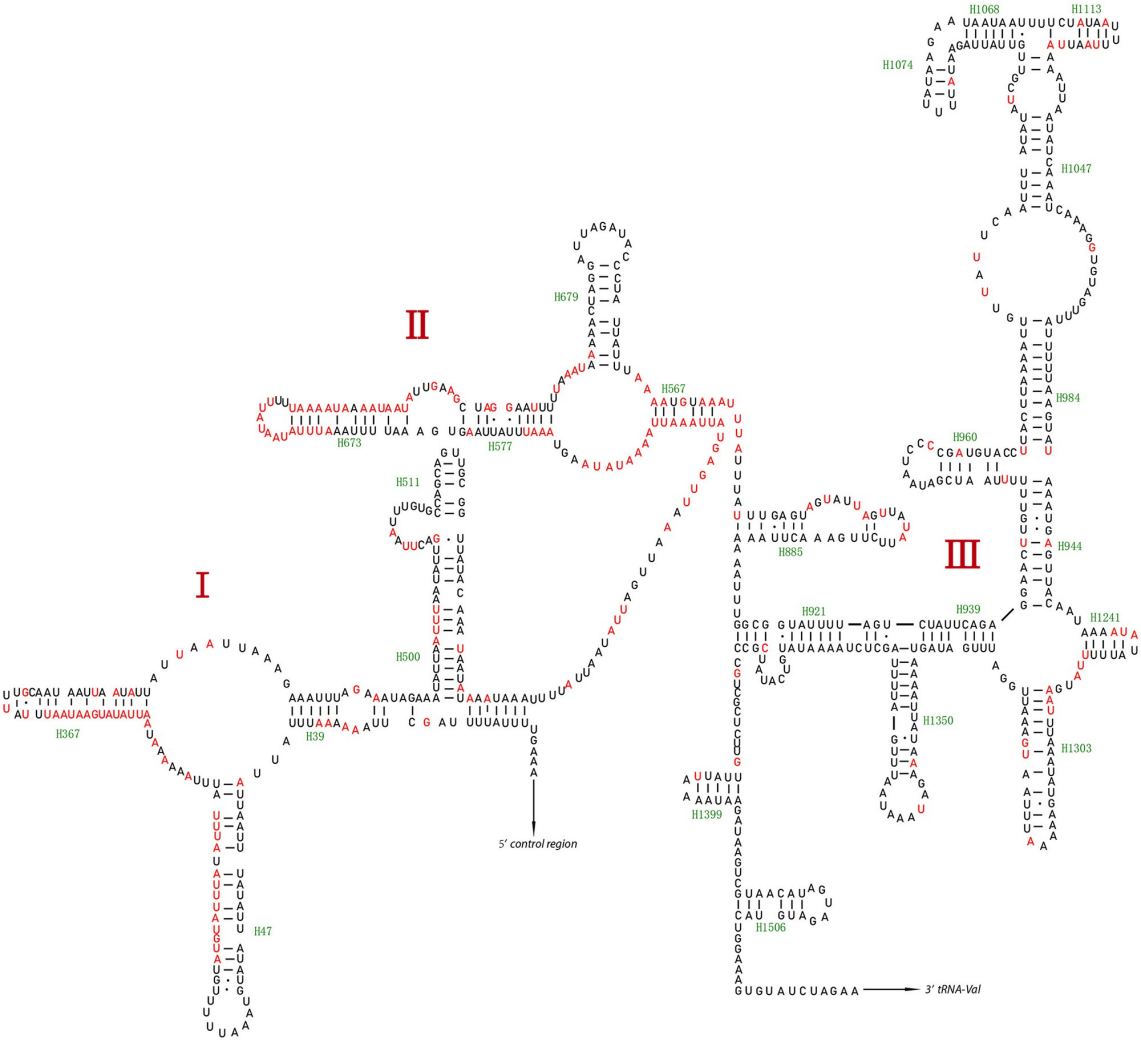
Predicated secondary structure of the 12S rRNA of the mitogenome of this study. Dashes (-) indicate Watson-Crick base pairing, GU pairs are connected by dots (·), and the variable sites are indicated in red.

*16S rRNAs* and *12S rRNAs* were both located in the N-strand. *16S rRNA* was located between *tRNA-L1* (UUR) and *tRNA-V*, and *12S rRNA* was between *tRNA-V* and the *D-loop* region. The secondary structure of *16S rRNA* was comprised of five domains (I, II, IV, V, and VI; domain III is absent in arthropods) and 44 helices (Fig 5), with domain IV showing higher sequence conservation than other domains (S19–S36 Figs). *12S rRNA* was comprised of three domains and 24 helices, with domain III showing the highest conservation (Fig 6 and S37–S54 Figs).

### Intergenic space and overlap region

Three conserved regions were found in the intergenic space and overlap regions of the Syrphidae mitogenomes. These were the overlap area between *COXI* and *Leu1* (TCTAA), the overlap area between *ATP8* and *ATP6* (ATGATAA), and one intergenic space between *ND1* and *Leu2* (AAAACAAG).

### D-loops

The *D-loops* of the Syrphidae mitogenomes have large variety, the length between 811 bp and 1410 bp. The gene of *D-loops* is the most important factors of the whole lengths of mitogenome in Syrphidae.

### Phylogenetic analysis

**Fig 7:**
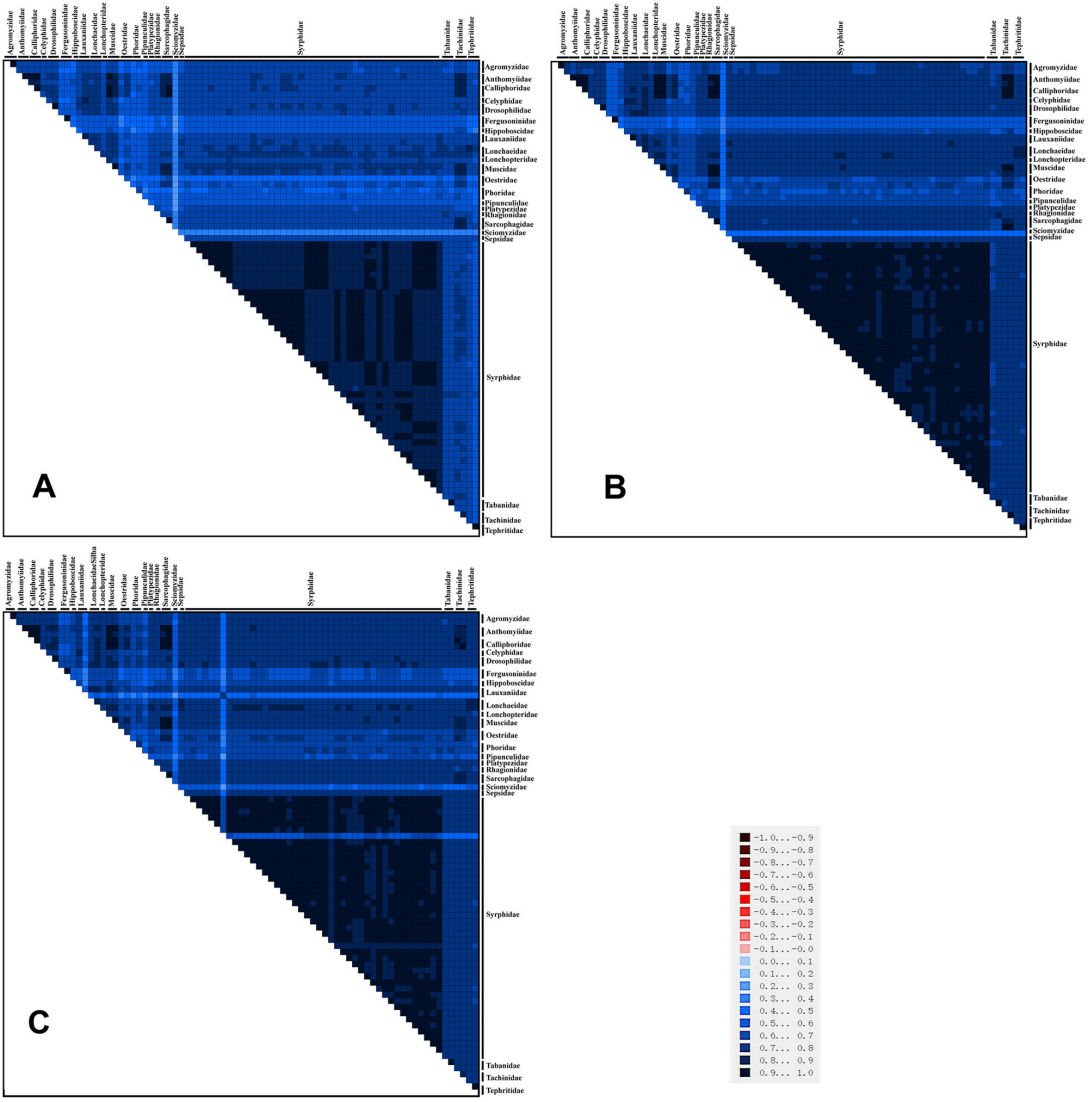
Heterogeneities of PCGA (A), PCG12(B) and PCG12RNA (C) in the mitogenomes of Muscomorpha. The mean similarity score between sequences is represented by color. The dark red (+1) to dark blue (-1) represent differences from heavy to light.

Detecting the base heterogeneity of mitogenome datasets used for constructing a phylogenetic tree is important, as strong heterogeneity can cause major errors [86–88]. On the basis of the calculation results obtained from the AliGROOVE [83], the heterogeneities of the PCG, PCG12, and PCG12RNA datasets were weak (Fig 7). Hence, the three datasets could be used to construct a phylogenetic tree.

**Fig 8:**
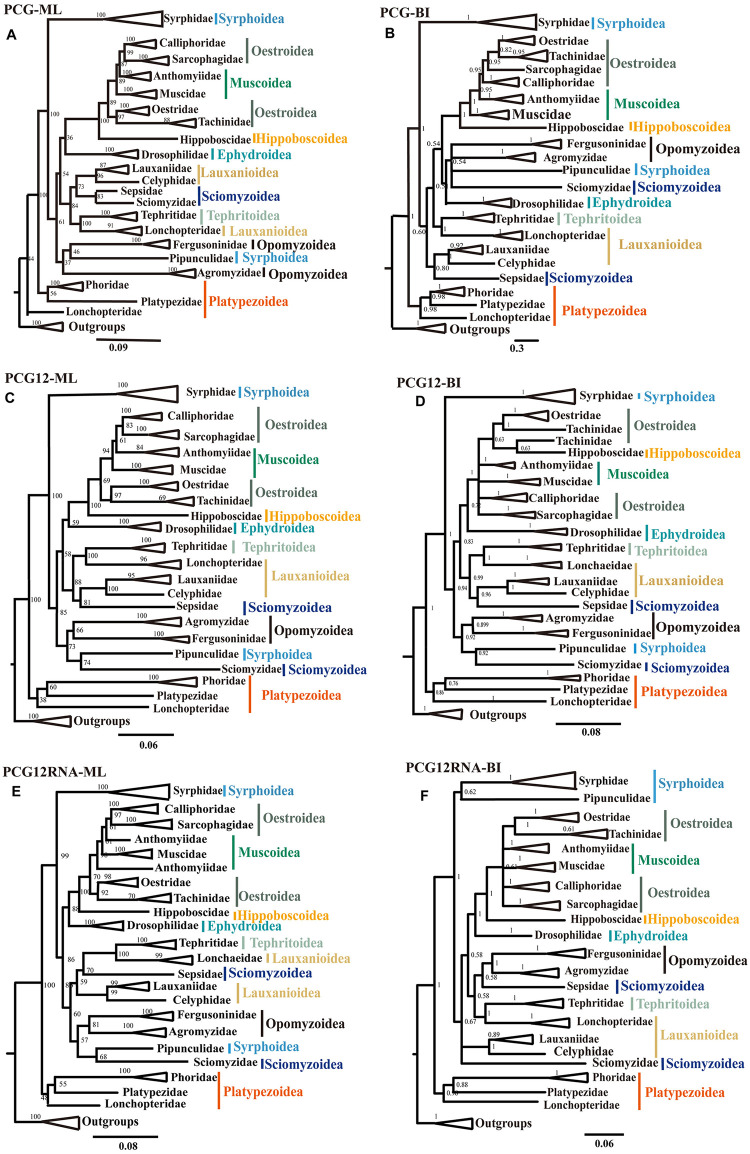
Phylogenetic trees of Muscomorpha inferred using Maximum likelihood (ML) and Bayesian inference (BI) analyses based on PCGs and rRNAs genes.

The ML and BI phylogenetic trees were reconstructed based on 76 Muscomorpha species and three outgroup species using three datasets to account for third codon compositional bias (PCG12RNA, PCG12, and PCGs), with the best partitioning scheme and models selected by PartitionFinder2. These six phylogenetic trees (three ML and three BI) had generally high support, with most nodes receiving high nodal support values and only a few nodes receiving moderate or low support (Figs 7 and 8).

In all resulting trees, all the Diptera families were monophyletic with strong support, with the exception of Anthomyiidae, which was paraphyletic in the PCG12RNA-ML tree (Fig 8E). Superfamilies were not necessarily monophyletic. The superfamily Syrphoidea was only monophyletic in the PCGRNA-BI tree; in other trees, *Pipunculus* sp. (Syrphoidea: Pipunculidae) always clustered with *Trypetoptera punctulata* (Sciomyzoidea: Sciomyzidae). Except in the PCG-ML and PCG12-BI trees, the superfamily Sciomyzoidea was paraphyletic, with *Nemopoda mamaevi* (Sciomyzoidea: Sepsidae) inferred as sister group to the Lauxanioidea. Oestroidea was always paraphyletic, with differing relationships between families depending on the dataset used (Fig 8).

**Fig 9:**
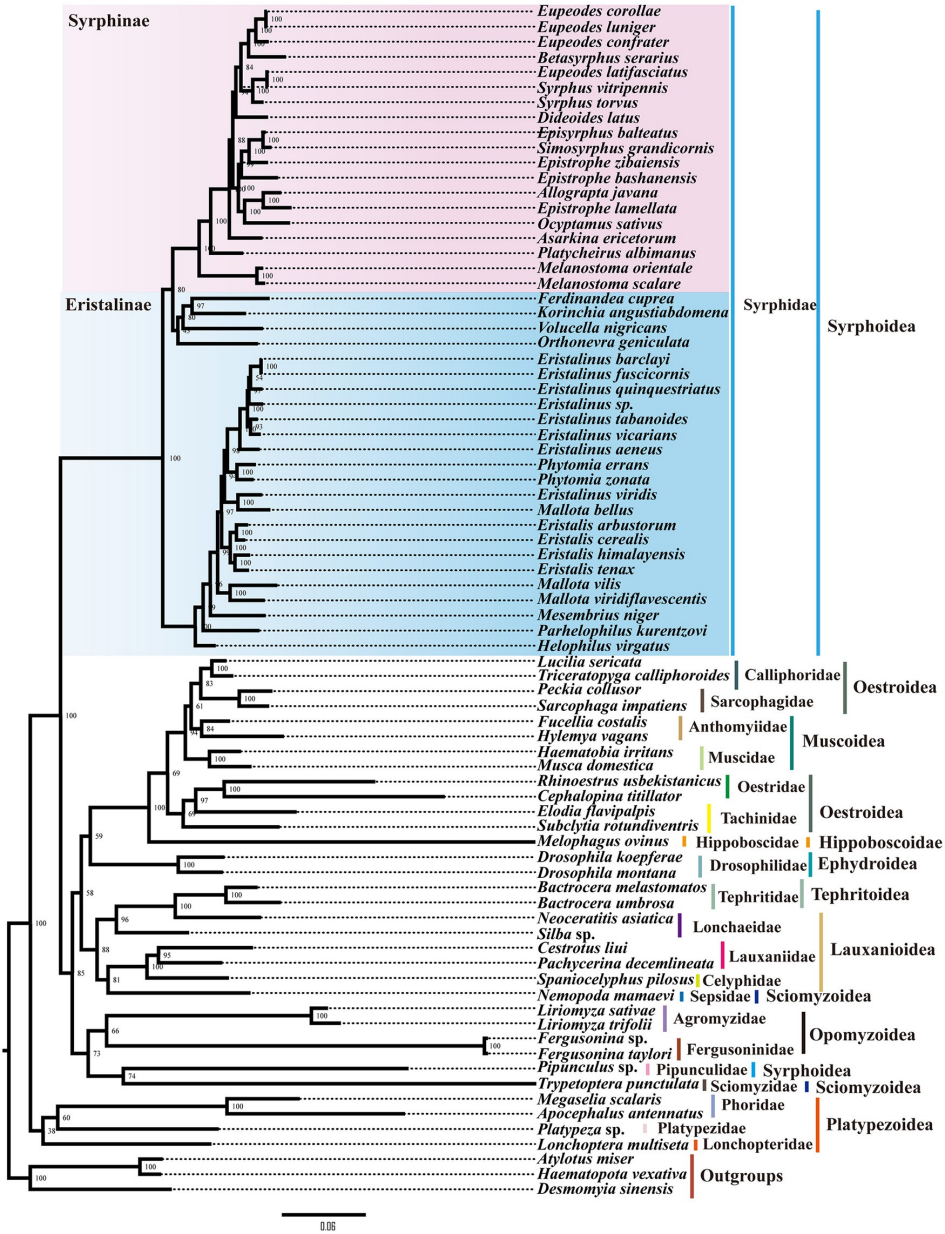
Phylogenetic tree of Muscomorpha inferred based on the first and second codons of the 13 PCGs using ML.

Both the BI and ML phylogenetic analyses of Syrphidae showed similar topology across all three datasets (Fig 9 and S55–S59 Figs). The monophyly of the subfamily Syrphinae was supported, within which the tribe Syrphini was clustered together with strong nodal supports, and the tribe Melanostomini was sister to Syrphini. The subfamily Eristalinae was paraphyletic in relation to Syrphinae. In particular, a monophyletic clade containing four putatively Eristalinae species appeared more closely related to the Syrphinae than the other Eristalinae, with the following topology: (((*Ferdinandea cuprea* + *Korinchia angustiabdomena*) + *Volucella nigricans*) + *Orthonevra geniculata*).

## Discussion

The mitogenomes from the 18 Syrphidae species in this study had similar genome length, genome structure, and nucleotide compositions as other Syrphidae mitogenomes, with many conserved regions. The results were in agreement with previous papers on Syrphidae, Diptera, and Insecta mitogenomes. The gene orders are similar with the arthropod ancestral mitochondrial genome. The conserved sequence (ATGATAA) in the overlap between *ATP8* and *ATP6* was found in other Muscomorpha insect mitogenomes, and is known from other insects, such as Fulgoroidae (Hemiptera) and Cercopidae (Hemiptera) [66,67]. This region was thought to be translated as a bicstron [68]. Those conserved fragment might play an important role in transcription and controlling the length of PCGs [69].

The lengths of every PCG in all 43 Syrphidae mitogenomes did not differ significantly, varying by no more than 35 bp, with the exception of *COXI* at 141 bp. This is expected from the relatively conserved characteristics of mitogenome PCGs. The limited length variations in all tRNAs and rRNAs observed among different species were mainly due to their stable secondary structures [89].

The most common nucleotide in the first codon position of the PCG start codon was A, followed by T in the second codon position. The stop codon was TA- or T—. The frequent use of these codons is an important reason for the A+T content being greater than the G+C content, as observed in other insect mitogenomes [90]. One reason for this base composition bias is asymmetric mutations that occurred during replication and transcription, and selective pressures after asymmetric mutations [17,74,91].

When these data were combined with other Muscomorpha mitogenomes, the resulting trees’ results agreed with some previous papers and disagreed with others. The choice of dataset and phylogenetic analysis method (ML or BI) affected some results. The PCG12-ML tree was most consistent with our point of view and previous morphological classifications about Syrphidae (Fig 9) [53,57].

Our results supported the paraphyly of Aschiza and Schizophora. The Platypezoidea diverged first, sister to the other species of Muscomorpha (Fig 8). The paraphyly of Syrphoidea due to Pipinculidae being closely related to the Schizophora and Opomyzoidea (Fig 8A–8E) supports previous findings [1,2,5].

The Acalyptratae was paraphyletic in our results. The superfamilies Ephydroidea, Tephritoidea, Lauxanioidea, and Opomyzoidea were monophyletic (Fig 8B, 8C, 8D and 8F). The superfamily Sciomyzoidea was paraphyletic. The family Sepsidae clustered with Lauxanioidea, and the Sciomyzidae was sister to Pipunculidae (Fig 8B–8D). The relationships of the Acalyptratae families remain obscure, likely because few characters were accumulated since the first Schizophora radiation, when these early lineages diverged rapidly [2]. More molecular information for the Acalyptratae is needed.

The monophyly of Calyptratea was supported. Its cluster formed almost as (Hippoboscoidea + (Oestroidea + (Muscoidea + Oestroidea))), and the family-level topology was always (Hippoboscidae + (Tachinidae + Oestridae) + (Anthomyiidae + (Muscoidae + (Sarcophagidae + Calliphoridae))))) (Fig 8A, 8C, 8E and 8F). Muscoidae was paraphyletic, as found in previous studies [2,11,35]. The monophyly of Oestroidea had been debated in previous mitogenome-based phylogenetic studies. Pu *et al*. [7], Wiegmann *et al*. [2], and Kutty *et al*. [11] concluded Oestroidea was monophyletic, while Li *et al*. [35] found Oestroidea was only monophyletic in PCG12RNA-BI and PCG12-BI trees, but paraphyletic in other datasets (PCG123, PCGRNA, PCG12-ML and PCG12RNA-ML). In our results, the superfamily Oestroidea was always paraphyletic, forming a monophyletic clade together with the superfamily Muscoidea [6,35]. This phenomenon may result from the limited mitogenomic data for these groups, so more mitogenome data for Oestroidea and Muscoidea could resolve this disagreement.

The phylogenetic analyses of Syrphidae found the subfamily Eristalinae was paraphyletic in relation to Syrphinae (Fig 9), in agreement with the results of Mengual *et al*. [51], Skevington and Yeates [8], and Young *et al*. [48]. Our results supported the monophyly of the tribe Eristalini in accordance with previous studies [9,51]. The taxonomic status of some species could not be resolved by these trees. For example, in the PCGs dataset (S55 and S56 Figs), *Asarkina ericetorum* was most closely related to *Dideoides latus*, while in other trees it was more closely related to *Ocyptamus sativus* and *Platycheirus albimanus*. Also, while the topology of (*Eristalinus viridis* + *Mallota bellus*) was well supported, the position of this clade was less stable (Fig 9). The genera of *Epistrophe*, *Eupeodes* and *Mallota* were not restored monophyletic in our study. The mitogenome information inferred in this study is limited, warranting the sampling of more taxa to confirm the conclusions reached here.

In this study, we found three unique, conserved fragments in all syrphid mitogenomes. Phylogenetic analyses were performed based on the nucleotide data of 13 PCGs and two rRNAs from 76 Muscomorpha and three outgroup species. Our results supported the paraphyly of Aschiza and Schizophora. The Acalyptratae was paraphyletic and the relationships of its superfamilies were difficult to determine. The monophyly of Calyptratea was supported, but the relationships of Oestroidea and Muscoidea need to be reconsidered. The monophyly of Syrphidae was supported. The Syrphinae were clustered into one branch, while the paraphyly of Eristalinae was strongly supported. We discussed the phylogenetic relationship of Muscomorpha using the latest comprehensive data, which we hope will provide a base reference for further phylogenetic analysis of Muscomorpha and the Diptera.

## Supporting information

S1 Fig Prediction of the secondary structure of tRNA of *Allograpta javana* in this study. The “-” indicates Watson-Crick base pairing and “•” indicates non-Watson-Crick base pairing.
(DOCX)

S2 Fig Prediction of the secondary structure of tRNA of *Asarkina ericetorum* in this study. The “-” indicates Watson-Crick base pairing and “•” indicates non-Watson-Crick base pairing.
(DOCX)

S3 Fig Prediction of the secondary structure of tRNA of *Betasyrphus serarius* in this study. The “-” indicates Watson-Crick base pairing and “•” indicates non-Watson-Crick base pairing.
(DOCX)

S4 Fig Prediction of the secondary structure of tRNA of *Dideoides latus* in this study. The “-” indicates Watson-Crick base pairing and “•” indicates non-Watson-Crick base pairing.
(DOCX)

S5 Fig Prediction of the secondary structure of tRNA of *Epistrophe bashanensis* in this study. The “-” indicates Watson-Crick base pairing and “•” indicates non-Watson-Crick base pairing.
(DOCX)

S6 Fig Prediction of the secondary structure of tRNA of *Epistrophe lamellata* in this study. The “-” indicates Watson-Crick base pairing and “•” indicates non-Watson-Crick base pairing.
(DOCX)

S7 Fig Prediction of the secondary structure of tRNA of *Epistrophe latifasciatus* in this study. The “-” indicates Watson-Crick base pairing and “•” indicates non-Watson-Crick base pairing.
(DOCX)

S8 Fig Prediction of the secondary structure of tRNA of *Epistrophe zibaiensis* in this study. The “-” indicates Watson-Crick base pairing and “•” indicates non-Watson-Crick base pairing.
(DOCX)

S9 Fig Prediction of the secondary structure of tRNA of *Eristalis arbustorum* in this study. The “-” indicates Watson-Crick base pairing and “•” indicates non-Watson-Crick base pairing.
(DOCX)

S10 Fig Prediction of the secondary structure of tRNA of *Eristalis himalayensis* in this study. The “-” indicates Watson-Crick base pairing and “•” indicates non-Watson-Crick base pairing.
(DOCX)

S11 Fig Prediction of the secondary structure of tRNA of *Eupeodes confrater* in this study. The “-” indicates Watson-Crick base pairing and “•” indicates non-Watson-Crick base pairing.
(DOCX)

S12 Fig Prediction of the secondary structure of tRNA of *Eupeodes luniger* in this study. The “-” indicates Watson-Crick base pairing and “•” indicates non-Watson-Crick base pairing.
(DOCX)

S13 Fig Prediction of the secondary structure of tRNA of *Mallota bellus* in this study. The “-” indicates Watson-Crick base pairing and “•” indicates non-Watson-Crick base pairing.
(DOCX)

S14 Fig Prediction of the secondary structure of tRNA of *Mallota vilis* in this study. The “-” indicates Watson-Crick base pairing and “•” indicates non-Watson-Crick base pairing.
(DOCX)

S15 Fig Prediction of the secondary structure of tRNA of *Mesembrius niger* in this study. The “-” indicates Watson-Crick base pairing and “•” indicates non-Watson-Crick base pairing.
(DOCX)

S16 Fig Prediction of the secondary structure of tRNA of *Mallota viridiflavescentis* in this study. The “-” indicates Watson-Crick base pairing and “•” indicates non-Watson-Crick base pairing.
(DOCX)

S17 Fig Prediction of the secondary structure of tRNA of *Parhelophilus kurentzovi* in this study. The “-” indicates Watson-Crick base pairing and “•” indicates non-Watson-Crick base pairing.
(DOCX)

S18 Fig Prediction of the secondary structure of tRNA of *Phytomia errans* in this study. The “-” indicates Watson-Crick base pairing and “•” indicates non-Watson-Crick base pairing.
(DOCX)

S19 Fig Prediction of the secondary structure of the *16S rRNA* of the *Allograpta javana* mitogenome. (DOCX)

S20 Fig Prediction of the secondary structure of the *16S rRNA* of the *Asarkina ericetorum* mitogenome. (DOCX)

S21 Fig Prediction of the secondary structure of the *16S rRNA* of the *Betasyrphus serarius* mitogenome. (DOCX)

S22 Fig Prediction of the secondary structure of the *16S rRNA* of the *Dideoides latus* mitogenome. (DOCX)

S23 Fig Prediction of the secondary structure of the *16S rRNA* of the *Epistrophe bashanensis* mitogenome. (DOCX)

S24 Fig Prediction of the secondary structure of the *16S rRNA* of the *Epistrophe lamellata* mitogenome. (DOCX)

S25 Fig Prediction of the secondary structure of the *16S rRNA* of the *Epistrophe latifasciatus* mitogenome. (DOCX)

S26 Fig Prediction of the secondary structure of the *16S rRNA* of the *Epistrophe zibaiensis* mitogenome. (DOCX)

S27 Fig Prediction of the secondary structure of the *16S rRNA* of the *Eristalis arbustorum* mitogenome. (DOCX)

S28 Fig Prediction of the secondary structure of the *16S rRNA* of the *Eristalis himalayensis* mitogenome. (DOCX)

S29 Fig Prediction of the secondary structure of the *16S rRNA* of the *Eupeodes confrater* mitogenome. (DOCX)

S30 Fig Prediction of the secondary structure of the *16S rRNA* of the *Eupeodes luniger* mitogenome. (DOCX)

S31 Fig Prediction of the secondary structure of the *16S rRNA* of the *Mallota bellus* mitogenome. (DOCX)

S32 Fig Prediction of the secondary structure of the *16S rRNA* of the *Mallota vilis* mitogenome. (DOCX)

S33 Fig Prediction of the secondary structure of the *16S rRNA* of the *Mesembrius niger* mitogenome. (DOCX)

S34 Fig Prediction of the secondary structure of the *16S rRNA* of the *Mallota viridiflavescentis* mitogenome. (DOCX)

S35 Fig Prediction of the secondary structure of the *16S rRNA* of the *Parhelophilus kurentzovi* mitogenome. (DOCX)

S36 Fig Prediction of the secondary structure of the *16S rRNA* of the *Phytomia errans* mitogenome. (DOCX)

S37 Fig Prediction of the secondary structure of the *16S rRNA* of the *Allograpta javana* mitogenome. (DOCX)

S38 Fig Prediction of the secondary structure of the *12S rRNA* of the *Asarkina ericetorum* mitogenome. (DOCX)

S39 Fig Prediction of the secondary structure of the *12S rRNA* of the *Betasyrphus serarius* mitogenome. (DOCX)

S40 Fig Prediction of the secondary structure of the *12S rRNA* of the *Dideoides latus* mitogenome. (DOCX)

S41 Fig Prediction of the secondary structure of the *12S rRNA* of the *Epistrophe bashanensis* mitogenome. (DOCX)

S42 Fig Prediction of the secondary structure of the *12S rRNA* of the *Epistrophe lamellata* mitogenome. (DOCX)

S43 Fig Prediction of the secondary structure of the *12S rRNA* of the *Epistrophe latifasciatus* mitogenome. (DOCX)

S44 Fig Prediction of the secondary structure of the *12S rRNA* of the *Epistrophe zibaiensis* mitogenome. (DOCX)

S45 Fig Prediction of the secondary structure of the *12S rRNA* of the *Eristalis arbustorum* mitogenome. (DOCX)

S46 Fig Prediction of the secondary structure of the *12S rRNA* of the *Eristalis himalayensis* mitogenome. (DOCX)

S47 Fig Prediction of the secondary structure of the *12S rRNA* of the *Eupeodes confrater* mitogenome. (DOCX)

S48 Fig Prediction of the secondary structure of the *12S rRNA* of the *Eupeodes luniger* mitogenome. (DOCX)

S49 Fig Prediction of the secondary structure of the *12S rRNA* of the *Mallota bellus* mitogenome. (DOCX)

S50 Fig Prediction of the secondary structure of the *12S rRNA* of the *Mallota vilis* mitogenome. (DOCX)

S51 Fig Prediction of the secondary structure of the *12S rRNA* of the *Mesembrius niger* mitogenome. (DOCX)

S52 Fig Prediction of the secondary structure of the *12S rRNA* of the *Mallota viridiflavescentis* mitogenome. (DOCX)

S53 Fig Prediction of the secondary structure of the *12S rRNA* of the *Parhelophilus kurentzovi* mitogenome. (DOCX)

S54 Fig Prediction of the secondary structure of the *12S rRNA* of the *Phytomia errans* mitogenome. (DOCX)

S55 Fig Phylogenetic tree of Muscomorpha. Inferred based on the 13 PCGs using ML.
(DOCX)

S56 Fig Phylogenetic tree of Muscomorpha. Inferred based on the 13 PCGs using BI.
(DOCX)

S57 Fig Phylogenetic tree of Muscomorpha. Inferred based on the first and second codons of the PCG12 using BI.
(DOCX)

S58 Fig Phylogenetic tree of Muscomorpha. Inferred based on the first and second codons of the 13 PCGs and 2 rRNAs using ML.
(DOCX)

S59 Fig Phylogenetic tree of Muscomorpha. Inferred based on the first and second codons of the 13 PCGs and 2 rRNAs using BI.
(DOCX)

S1 Table Gene organization of the complete mitogenome of *Allograpta javana*. (DOCX)

S2 Table Gene organization of the complete mitogenome of *Asarkina ericetorum*. (DOCX)

S3 Table Gene organization of the complete mitogenome of *Betasyrphus serarius*. (DOCX)

S4 Table Gene organization of the complete mitogenome of *Dideoides latus*. (DOCX)

S5 Table Gene organization of the complete mitogenome of *Epistrophe bashanensis*. (DOCX)

S6 Table Gene organization of the complete mitogenome of *Epistrophe lamellate*. (DOCX)

S7 Table Gene organization of the complete mitogenome of *Epistrophe latifasciatus*. (DOCX)

S8 Table Gene organization of the complete mitogenome of *Epistrophe zibaiensis*. (DOCX)

S9 Table Gene organization of the complete mitogenome of *Eristalis arbustorum*. (DOCX)

S10 Table Gene organization of the complete mitogenome of *Eristalis himalayensis*. (DOCX)

S11 Table Gene organization of the complete mitogenome of *Eupeodes confrater*. (DOCX)

S12 Table Gene organization of the complete mitogenome of *Eupeodes luniger*. (DOCX)

S13 Table Gene organization of the complete mitogenome of *Mallota bellus*. (DOCX)

S14 Table Gene organization of the complete mitogenome of *Mallota vilis*. (DOCX)

S15 Table Gene organization of the complete mitogenome of *Mesembrius niger*. (DOCX)

S16 Table Gene organization of the complete mitogenome of *Mallota viridiflavescentis*. (DOCX)

S17 Table Gene organization of the complete mitogenome of *Parhelophilus kurentzovi*. (DOCX)

S18 Table Gene organization of the complete mitogenome of *Phytomia errans*. (DOCX)
