# Supplementary material for: Eighteen mitochondrial genomes of Syrphidae (Insecta: Diptera: Brachycera) with a phylogenetic analysis of Muscomorpha
Source: PLoS One. 2023 Jan 5;18(1):e0278032. doi: 10.1371/journal.pone.0278032 (PMC9815649; doi:10.1371/journal.pone.0278032)
Supplement: S28 Fig — (DOCX) [file pone.0278032.s028.docx]

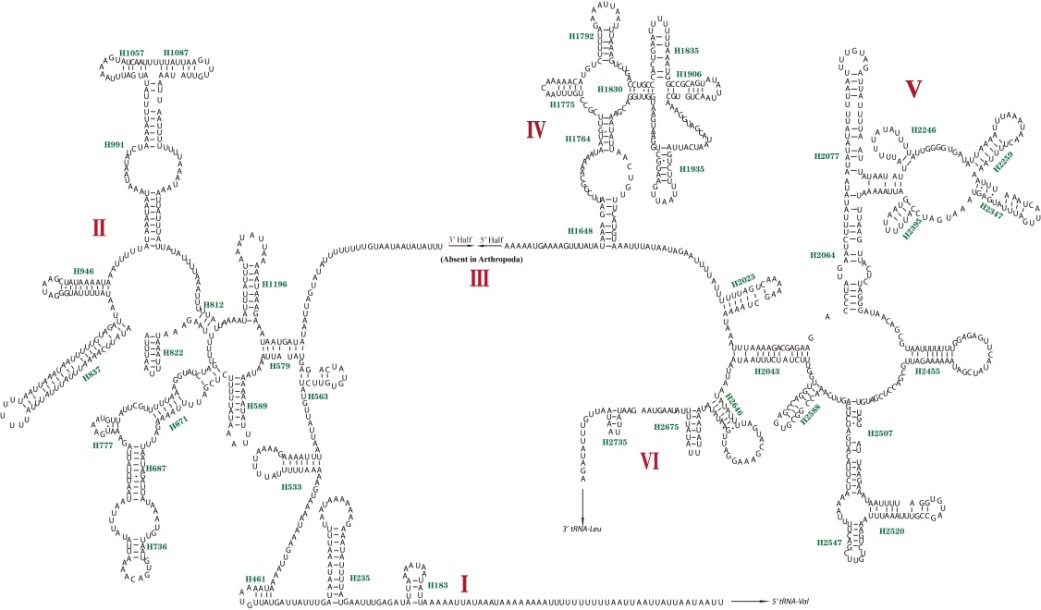


**Supplementary Figure 28.** Prediction of the secondary structure of *16S rRNA* of *Eristalis himalayensis* mitogenome.
