# Supplementary material for: Eighteen mitochondrial genomes of Syrphidae (Insecta: Diptera: Brachycera) with a phylogenetic analysis of Muscomorpha
Source: PLoS One. 2023 Jan 5;18(1):e0278032. doi: 10.1371/journal.pone.0278032 (PMC9815649; doi:10.1371/journal.pone.0278032)
Supplement: S50 Fig — (DOCX) [file pone.0278032.s050.docx]

**
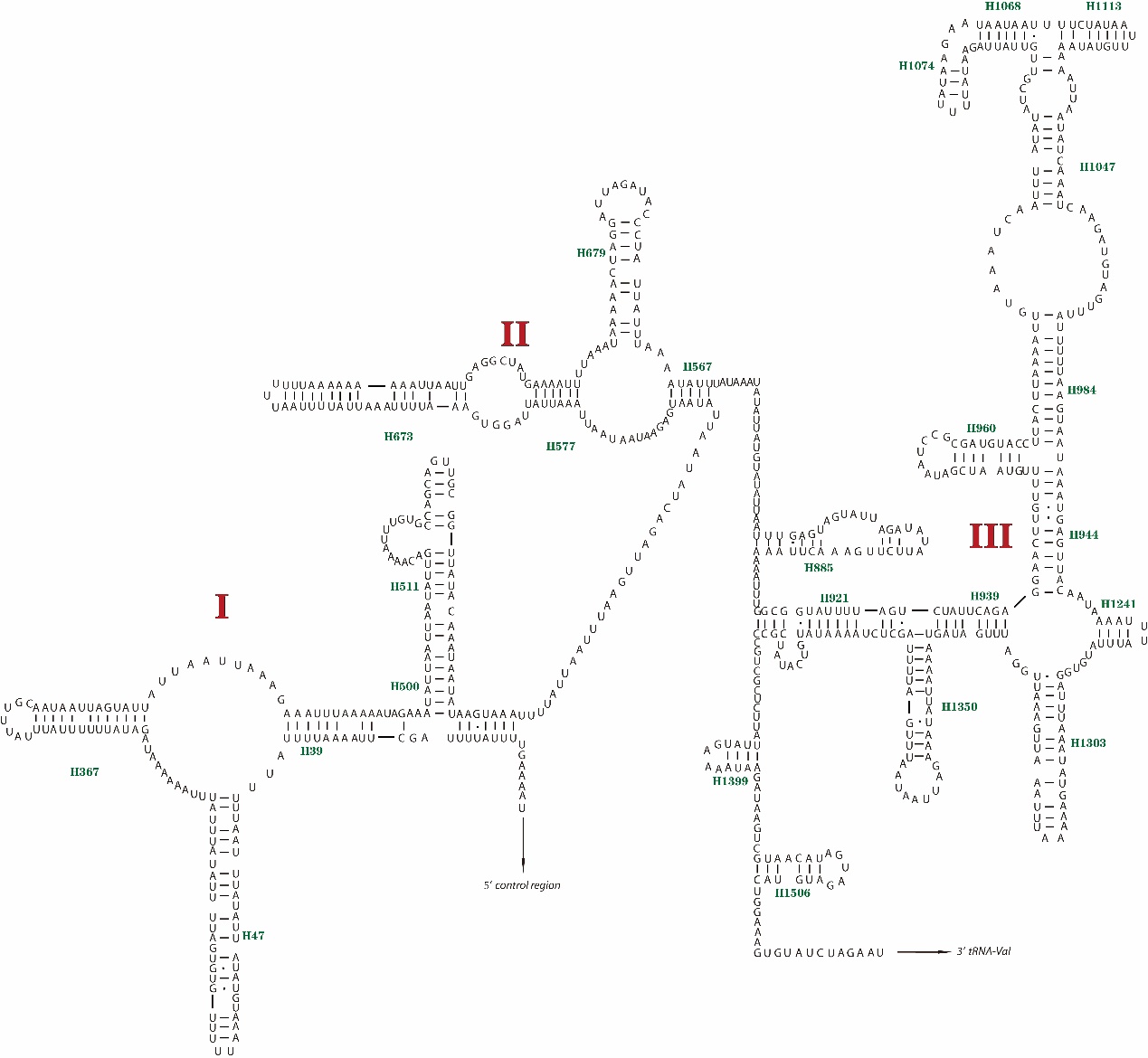
**

**Supplementary Figure 50.** Prediction of the secondary structure of *12S rRNA* of *Mallota vilis* mitogenome.
