# Supplementary figures and images for: Eighteen mitochondrial genomes of Syrphidae (Insecta: Diptera: Brachycera) with a phylogenetic analysis of Muscomorpha
Source: PLoS One. 2023 Jan 5;18(1):e0278032. doi: 10.1371/journal.pone.0278032 (PMC9815649; doi:10.1371/journal.pone.0278032)

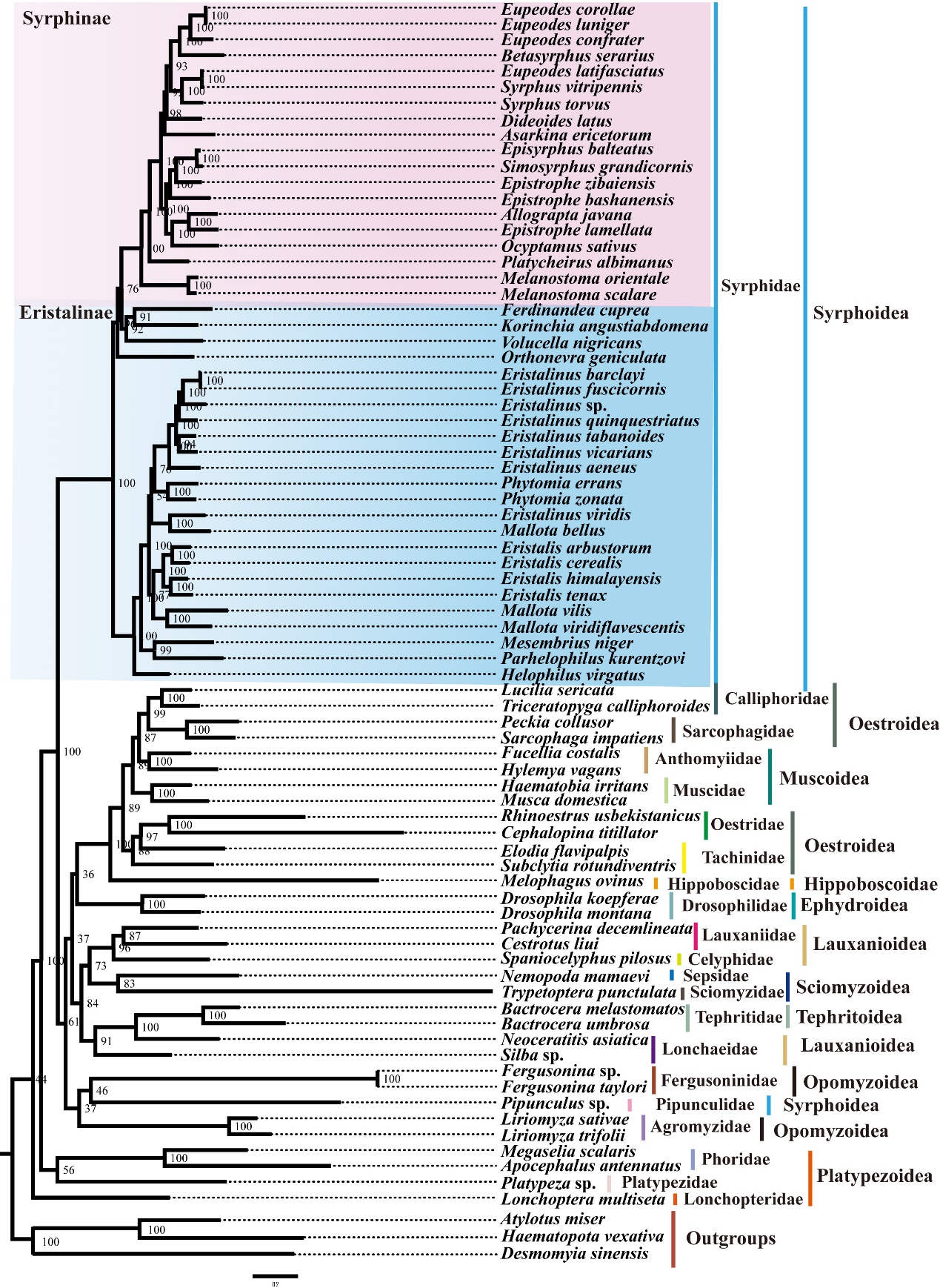


**Supplementary Figure 55.** Phylogenetic tree of Muscomorpha*.* Inferred based on the13 PCGs using ML.

Supplement: S55 Fig — Inferred based on the 13 PCGs using ML. (DOCX) [file pone.0278032.s055.docx]

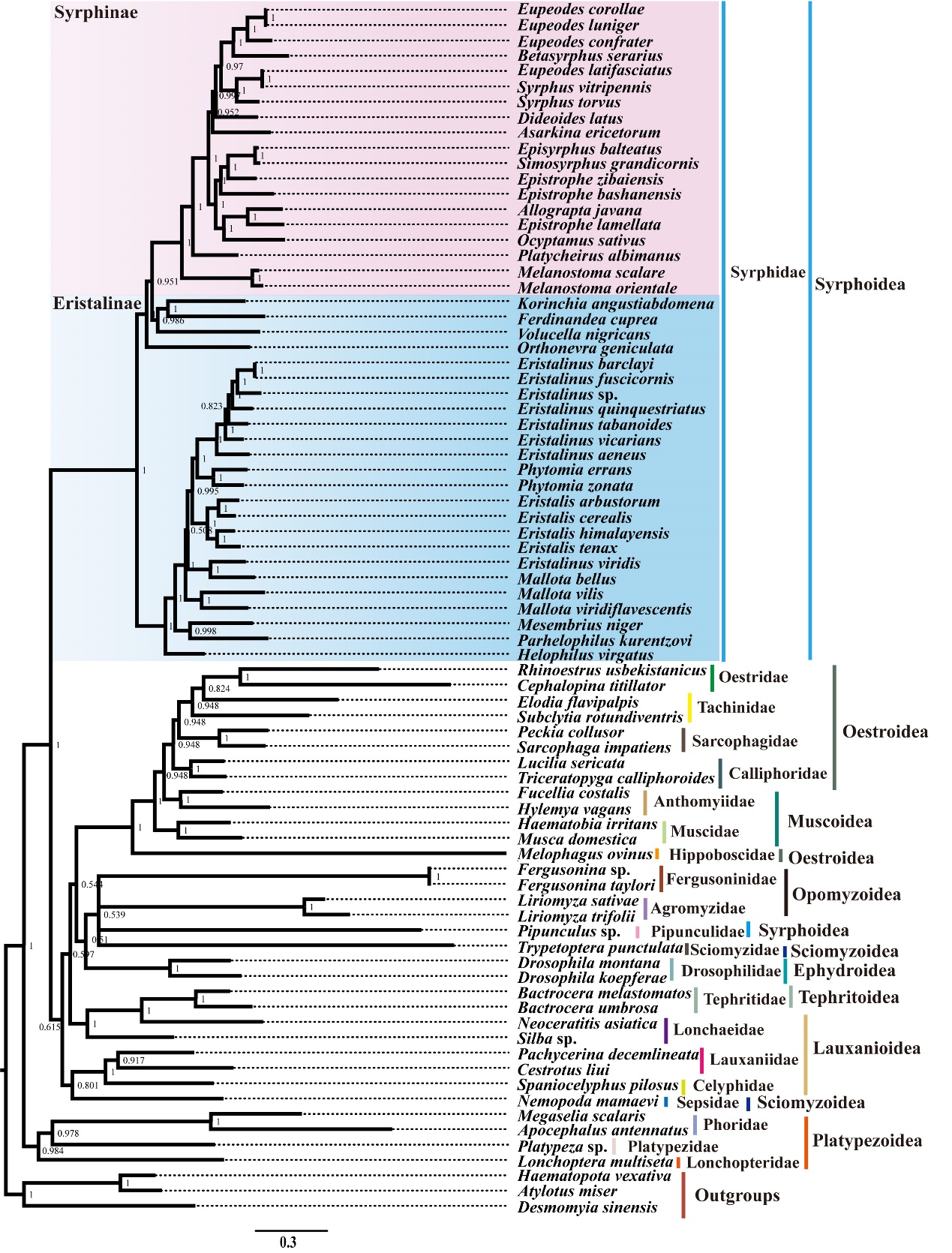


**Supplementary Figure 56.** Phylogenetic tree of Muscomorpha*.* Inferred based on the 13 PCGs using BI.

Supplement: S56 Fig — Inferred based on the 13 PCGs using BI. (DOCX) [file pone.0278032.s056.docx]
