# Supplementary material for: Eighteen mitochondrial genomes of Syrphidae (Insecta: Diptera: Brachycera) with a phylogenetic analysis of Muscomorpha
Source: PLoS One. 2023 Jan 5;18(1):e0278032. doi: 10.1371/journal.pone.0278032 (PMC9815649; doi:10.1371/journal.pone.0278032)
Supplement: S57 Fig — Inferred based on the first and second codons of the PCG12 using BI. (DOCX) [file pone.0278032.s057.docx]

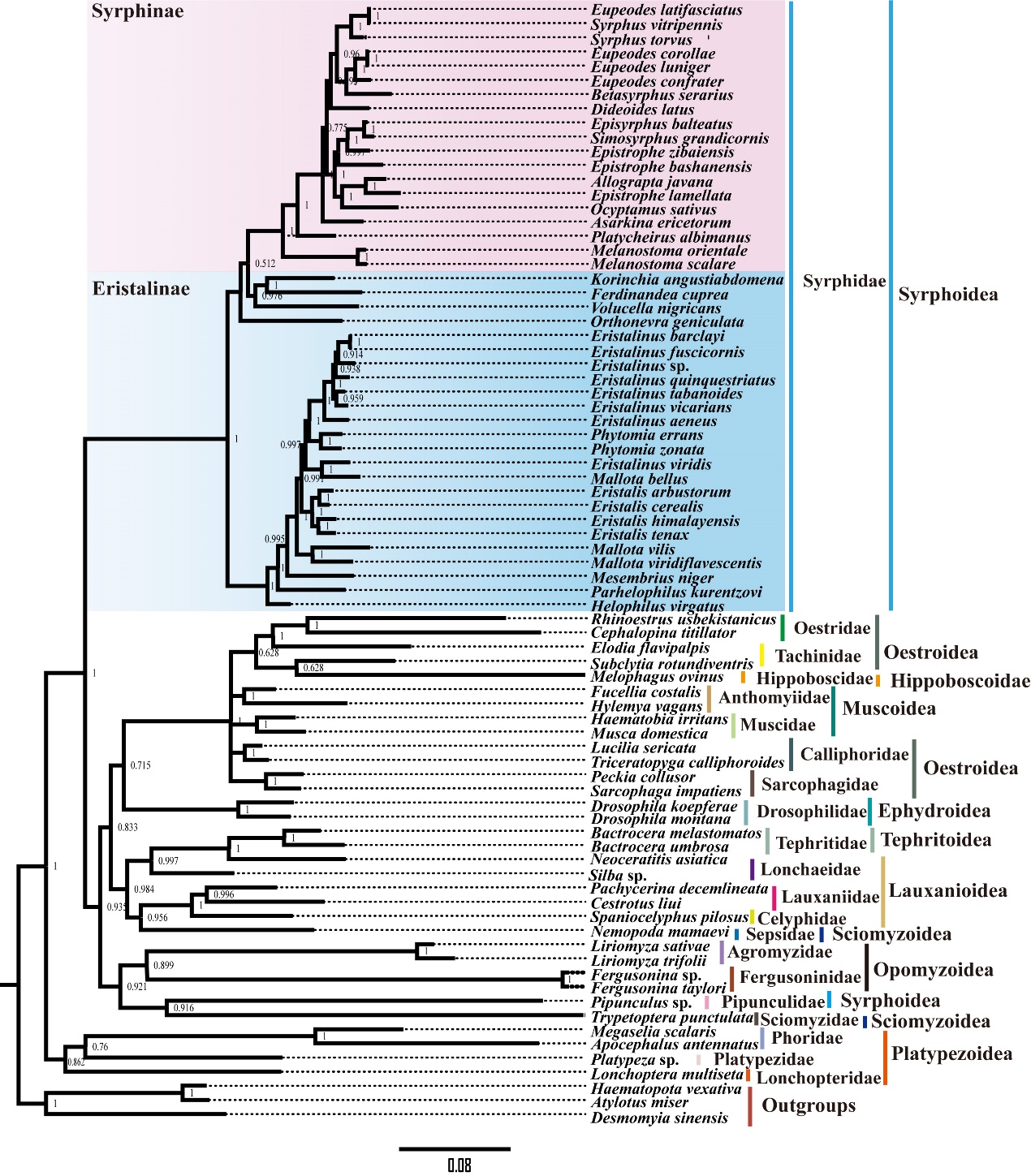
**Supplementary Figure 57.** Phylogenetic tree of Muscomorpha*.* Inferred based on the first and second codons of the 13 PCGs using BI.
