# Supplementary material for: Eighteen mitochondrial genomes of Syrphidae (Insecta: Diptera: Brachycera) with a phylogenetic analysis of Muscomorpha
Source: PLoS One. 2023 Jan 5;18(1):e0278032. doi: 10.1371/journal.pone.0278032 (PMC9815649; doi:10.1371/journal.pone.0278032)
Supplement: S1 Table — (DOCX) [file pone.0278032.s060.docx]

**Supplementary** **Table 1** Gene organization of the complete mitogenome of *Allograpta javana*

| Gene | Direction | Location | Size (bp) | Start/stop codon | Anticodon | Intergennic nucleotide |
| --- | --- | --- | --- | --- | --- | --- |
| *trn-l* | F | 1-66 | 66 |  | 30-32/GAT |  |
| *trn-Q* | R | 68-136 | 69 |  | 104-106/GTT | 1 |
| *trn-M* | F | 207-275 | 69 |  | 237 - 239/CAT | 70 |
| *nad2* | F | 276-1310 | 1035 | ATT/TAA |  | 0 |
| *trn-W* | F | 1,309-1,376 | 68 |  | 1,340-1,342/TCA | -2 |
| *trn-C* | R | 1,385-1,450 | 66 |  | 1352-1354/GCA | 8 |
| *trn-Y* | R | 1,455-1,521 | 67 |  | 1,488-1,490 /GTA | 4 |
| *cox1* | F | 1,572-3,110 | 1,539 | TTG/TAA |  | 50 |
| *trn-L1* | F | 3,106-3,171 | 66 |  | 3,135-3,137/TAA | -5 |
| *cox2* | F | 3,183-3,866 | 684 | ATG/TAA |  | 11 |
| *trn-K* | F | 3,867-3,937 | 71 |  | 3,897-3,899/CTT | 0 |
| *trn-D* | F | 3,970-4,036 | 67 |  | 4,001-4,003/GTC | 32 |
| *atp8* | F | 4,037-4,198 | 162 | ATT/TAA |  | 0 |
| *atp6* | F | 4,192-4,869 | 678 | ATG/TAA |  | -7 |
| *cox3* | F | 4,882-5,670 | 789 | ATG/TAA |  | 12 |
| *trn-G* | F | 5,683-5,748 | 66 |  | 5610-5612TCC | 12 |
| *nad3* | F | 5,749-6,102 | 354 | ATT/TAA |  | 0 |
| *trn-A* | F | 6,105-6,171 | 67 |  | 6,136-6,138/TGC | 2 |
| *trn-R* | F | 6,171-6,234 | 64 |  | 6,200-6,202/TCG | -1 |
| *trn-N* | F | 6,239-6,305 | 67 |  | 6,270-6,272/GTT | 4 |
| *trn-S* | F | 6,306-6,372 | 67 |  | 6,331-6,333/GCT | 0 |
| *trn-E* | F | 6,374-6,440 | 67 |  | 6,404-6,406/TTC | 1 |
| *trn-F* | R | 6,460-6,526 | 67 |  | 6415-6417/GAA | 19 |
| *nad5* | R | 6,526-8,265 | 1,740 | ATT/TAA |  | -1 |
| *trn-H* | R | 8,263-8,329 | 67 |  | 8,219-8,221/GTG | -3 |
| *nad4* | R | 8,329-9,669 | 1,341 | ATG/TAA |  | -1 |
| *nad4L* | R | 9,663-9,959 | 297 | ATG/TAA |  | -7 |
| *trn-T* | F | 9,962-10,027 | 66 |  | 9,992-9,994/TGT | 2 |
| *trn-P* | R | 10,028-10,093 | 66 |  | 10,061-10,063/TGG | 0 |
| *nad6* | F | 10,096-10,620 | 525 | ATT/TAA |  | 2 |
| *cob* | F | 10,629-11,765 | 1,137 | ATG/TAA |  | 7 |
| *trn-S2* | F | 11,769-11,836 | 68 |  | 11,798-11,800/TGA | 3 |
| *nad1* | R | 11,853-12,791 | 939 | ATA/TAA |  | 16 |
| *trn-L2* | R | 12,802-12,866 | 65 |  | 12,835-12,837/TAG | 10 |
| *rrnL-16S* | R | 12,867-14,200 | 1,334 |  |  | 0 |
| *trn-V* | R | 14,201-14,272 | 72 |  | 14,237-14,239/TAC | 0 |
| *rrnS-12S* | R | 14,274-15,069 | 796 |  |  | 0 |
| *D-loop* |  | 15,070-16,387 | 1,318 |  |  | 0 |
