# Supplementary material for: Eighteen mitochondrial genomes of Syrphidae (Insecta: Diptera: Brachycera) with a phylogenetic analysis of Muscomorpha
Source: PLoS One. 2023 Jan 5;18(1):e0278032. doi: 10.1371/journal.pone.0278032 (PMC9815649; doi:10.1371/journal.pone.0278032)
Supplement: S2 Table — (DOCX) [file pone.0278032.s061.docx]

**Supplementary Table 2** Gene organization of the complete mitogenome of *Asarkina ericetorum*

| Gene | Direction | Location | Size (bp) | Start/stop codon | Anticodon | Intergennic nucleotide |
| --- | --- | --- | --- | --- | --- | --- |
| *trn-l* | F | 1-67 | 67 |  | 31-33/GAT |  |
| *trn-Q* | R | 65-133 | 69 |  | 101-103/TTG | -3 |
| *trn-M* | F | 145-213 | 69 |  | 175-177/CAT | 11 |
| *nad2* | F | 214-1,251 | 1,038 | ATT/TAA |  | 0 |
| *trn-W* | F | 1,250-1,317 | 68 |  | 1,281-1,283/TCA | -2 |
| *trn-C* | R | 1,325-1,390 | 66 |  | 1,359-1,361/GCA | 7 |
| *trn-Y* | R | 1,404-1,471 | 68 |  | 1,459-1,461/GTA | 13 |
| *cox1* | F | 1,471-3,024 | 1,578 | ATA/TAA |  | -1 |
| *trn-L1* | F | 3,020-3,085 | 66 |  | 3,049-3,051/TAA | -5 |
| *cox2* | F | 3,088-3,771 | 684 | ATG/TAA |  | 2 |
| *trn-K* | F | 3,773-3,843 | 71 |  | 3,803-3,805/CTT | 1 |
| *trn-D* | F | 3,853-3,917 | 65 |  | 3,884-3,886/GTC | 9 |
| *atp8* | F | 3,920-4,081 | 162 | ATT/TAA |  | 2 |
| *atp6* | F | 4,075-4,752 | 678 | ATG/TAA |  | -7 |
| *cox3* | F | 4,773-5,561 | 789 | ATG/TAA |  | 20 |
| *trn-G* | F | 5,565-5,629 | 65 |  | 5,594-5,596/TCC | 3 |
| *nad3* | F | 5,630-5,983 | 354 | ATT/TAG |  | 0 |
| *trn-A* | F | 5,987-6,055 | 69 |  | 6,018-6,020/TGC | 3 |
| *trn-R* | F | 6,055-6,118 | 64 |  | 6,084-6,086/TCG | -1 |
| *trn-N* | F | 6,123-6,189 | 67 |  | 6,123-6,189/GTT | 4 |
| *trn-S* | F | 6,190-6,256 | 67 |  | 6,215-6,217/GCT | 0 |
| *trn-E* | F | 6,257-6,322 | 66 |  | 6,287-6,289/TTC | 0 |
| *trn-F* | R | 6,344-6,409 | 66 |  | 6,375-6,377/GAA | 21 |
| *nad5* | R | 6,410-8,131 | 1,722 | ATT/TAA |  | 0 |
| *trn-H* | R | 8,147-8,212 | 66 |  | 8,180-8,182/GTG | 15 |
| *nad4* | R | 8,213-9,553 | 1,341 | ATG/TAA |  | 0 |
| *nad4L* | R | 9,547-9,843 | 297 | ATG/TAA |  | -7 |
| *trn-T* | F | 9,846-9,911 | 66 |  | 9,876-9,878/TGT | 2 |
| *trn-P* | R | 9,912-9,977 | 66 |  | 9,945-9,947/TGG | 0 |
| *nad6* | F | 9,980-10,504 | 525 | ATT/TAA |  | 2 |
| *cob* | F | 10,508-11,644 | 1,137 | ATG/TAA |  | 3 |
| *trn-S2* | F | 11,650-11,717 | 68 |  | 11,679-11,681/TGA | 5 |
| *nad1* | R | 11,734-12,672 | 939 | ATA/TAA |  | 16 |
| *trn-L2* | R | 12,683-12,747 | 65 |  | 12,716-12,718/TAG | 10 |
| *rrnL-16S* | R | 12,833-14,172 | 1,338 |  |  | 0 |
| *trn-V* | R | 14,086-14,157 | 72 |  | 14,122-14,124/TAC | 0 |
| *rrnS-12S* | R | 14,158-14,954 | 797 |  |  | 0 |
| *D-loop* |  | 14,955-16,080 | 1,126 |  |  | 0 |
