# Supplementary material for: Eighteen mitochondrial genomes of Syrphidae (Insecta: Diptera: Brachycera) with a phylogenetic analysis of Muscomorpha
Source: PLoS One. 2023 Jan 5;18(1):e0278032. doi: 10.1371/journal.pone.0278032 (PMC9815649; doi:10.1371/journal.pone.0278032)
Supplement: S3 Table — (DOCX) [file pone.0278032.s062.docx]

**Supplementary** **Table 3** Gene organization of the complete mitogenome of *Betasyrphus serarius*

| Gene | Direction | Location | Size (bp) | Start/stop codon | Anticodon | Intergennic nucleotide |
| --- | --- | --- | --- | --- | --- | --- |
| *trn-l* | F | 1-67 | 67 |  | 30-32/GAT |  |
| *trn-Q* | R | 65-133 | 69 |  | 103-105/TTG | -3 |
| *trn-M* | F | 144-212 | 69 |  | 174-176/CAT | 10 |
| *nad2* | F | 213-12,41 | 1,029 | ATA/TAA |  | 0 |
| *trn-W* | F | 1,241-1,308 | 68 |  | 1,272-1,274/TCA | -1 |
| *trn-C* | R | 1,318-1,387 | 70 |  | 1,353-1,355/GCA | 9 |
| *trn-Y* | R | 1,409-1,474 | 66 |  | 1,441-1,443/GTA | 21 |
| *cox1* | F | 1,476-3,032 | 1,557 | ATA/TAA |  | 1 |
| *trn-L1* | F | 3,028-3,093 | 66 |  | 3,057-3,059/TAA | -5 |
| *cox2* | F | 3,096-3,779 | 684 | ATG/TAA |  | 2 |
| *trn-K* | F | 3,780 -3,850 | 71 |  | 3,810-3,812/CTT | 0 |
| *trn-D* | F | 3,871-3,937 | 67 |  | 3,902-3,904/GTC | 20 |
| *atp8* | F | 3,938-4,099 | 162 | ATT/TAA |  | 0 |
| *atp6* | F | 4093-4770 | 678 | ATG/TAA |  | -7 |
| *cox3* | F | 4,785-5,573 | 789 | ATG/TAA |  | 14 |
| *trn-G* | F | 5,577-5,642 | 66 |  | 5,606-5,608/TCC | 2 |
| *nad3* | F | 5,643-5,996 | 354 | ATT/TAA |  | -3 |
| *trn-A* | F | 5,999-6,066 | 68 |  | 6,029-6,031/TGC | 2 |
| *trn-R* | F | 6,066-6,129 | 64 |  | 6,095-6,097TCG | -1 |
| *trn-N* | F | 6,163-6,228 | 66 |  | 6,194-6,196 GTT | 33 |
| *trn-S* | F | 6,228-6,296 | 69 |  | 6,254-6,256/GCT | -1 |
| *trn-E* | F | 6,300-6,366 | 67 |  | 6,330-6,332/TTC | 3 |
| *trn-F* | R | 6,460-6,395 | 66 |  | 6,426-6,428/GAA | 28 |
| *nad5* | R | 6,459-8,180 | 1,722 | ATT/TAT |  | -2 |
| *trn-H* | R | 8,196-8,262 | 67 |  | 8,229-8,231/GTG | 15 |
| *nad4* | R | 8,262-9,602 | 1,341 | ATG/TAA |  | -1 |
| *nad4L* | R | 9,596-9,892 | 297 | ATG/TAA |  | 7 |
| *trn-T* | F | 9,895-9,961 | 67 |  | 9,925-9,927/TGT | 2 |
| *trn-P* | R | 9,962-10,027 | 66 |  | 9,995-9,997/TGG | -1 |
| *nad6* | F | 10,030-10,554 | 525 | ATT/TAA |  | 2 |
| *cob* | F | 10,558-11,694 | 1,137 | ATG/TAA |  | 11 |
| *trn-S2* | F | 11,698-11,765 | 68 |  | 11,727-11,729/TGA | 3 |
| *nad1* | R | 11,782-12,720 | 939 | ATA/TAG |  | 16 |
| *trn-L2* | R | 12,731-12,795 | 65 |  | 12,764-12,766/TAG | 1 |
| *rrnL-16S* | R | 12,796-14,127 | 1,331 |  |  | 0 |
| *trn-V* | R | 14,128-14,199 | 73 |  | 14,164-14,166/TAC | 0 |
| *rrnS-12S* | R | 14,200-14,988 | 789 |  |  | 0 |
| *D-loop* |  | 14,989-16,369 | 1,380 |  |  | 0 |
