# Supplementary material for: Eighteen mitochondrial genomes of Syrphidae (Insecta: Diptera: Brachycera) with a phylogenetic analysis of Muscomorpha
Source: PLoS One. 2023 Jan 5;18(1):e0278032. doi: 10.1371/journal.pone.0278032 (PMC9815649; doi:10.1371/journal.pone.0278032)
Supplement: S4 Table — (DOCX) [file pone.0278032.s063.docx]

**Supplementary Table 4** Gene organization of the complete mitogenome of *Dideoides latus*

| Gene | Direction | Location | Size | Start/stop codon | Anticodon | Intergennic nucleotide |
| --- | --- | --- | --- | --- | --- | --- |
| *trn-l* | F | 1-67 | 67 |  | 31-33/GAT |  |
| *trn-Q* | R | 65-133 | 69 |  | 101-103/TTG | 27 |
| *trn-M* | F | 160-228 | 69 |  | 190-192/CAT | 1 |
| *nad2* | F | 229-1,257 | 1,029 | ATT/TAA |  | 5 |
| *trn-W* | F | 1,262-1,330 | 69 |  | 1,294-1,296/TCA | 4 |
| *trn-C* | R | 1,334-1,399 | 66 |  | 1,367-1,370/GCA | 4 |
| *trn-Y* | R | 1,403-1,477 | 75 |  | 1,444-1,446/GTA | 38 |
| *cox1* | F | 1,515-3,053 | 1,614 | TTG/TAA |  | -4 |
| *trn-L1* | F | 3,049-3,114 | 66 |  | 3,078-3,080/TAA | 3 |
| *cox2* | F | 3,103-3,789 | 684 | ATG/TAA |  | 2 |
| *trn-K* | F | 3,802-3,872 | 71 |  | 3,832-3,835/CTT | 33 |
| *trn-D* | F | 3,905-3,971 | 67 |  | 3,936-3,939/GTC | 1 |
| *atp8* | F | 3,972-4,133 | 162 | ATC/TAA |  | -5 |
| *atp6* | F | 4,127-4,804 | 678 | ATG/TAA |  | 5 |
| *cox3* | F | 4,809-5,597 | 789 | ATG/TAA |  | 5 |
| *trn-G* | F | 5,602-5,670 | 69 |  | 5/631-5,633/TCC | 1 |
| *nad3* | F | 5,671-6,024 | 354 | ATT/TAA |  | 4 |
| *trn-A* | F | 6,028-6,097 | 70 |  | 6,058-6,060/TGC | 0 |
| *trn-R* | F | 6,097-6,161 | 65 |  | 6,127-6,129/TCG | 5 |
| *trn-N* | F | 6,166-6,231 | 66 |  | 6,197-6,199/GTT | 1 |
| *trn-S* | F | 6,232-6,298 | 67 |  | 6,257-6,259/GCT | 5 |
| *trn-E* | F | 6,303-6,367 | 65 |  | 6,331-6,334/TTC | 50 |
| *trn-F* | R | 6,417-6,483 | 67 |  | 6,448-6,451/GAA | 1 |
| *nad5* | R | 6,484-8,223 | 1,740 | ATT/TAA |  | -2 |
| *trn-H* | R | 8,221-8,286 | 66 |  | 8,254-8,256/GTG | 1 |
| *nad4* | R | 8,287-9,627 | 1,341 | ATG/TAA |  | -6 |
| *nad4L* | R | 9,621-9,917 | 297 | ATG/TAA |  | 3 |
| *trn-T* | F | 9,920-9,984 | 65 |  | 9,949-9,952/TGT | 1 |
| *trn-P* | R | 9,985-10,050 | 66 |  | 10,018-10,021/TGG | 3 |
| *nad6* | F | 10,053-10,577 | 525 | ATT/TAA |  | 7 |
| *cob* | F | 10,584-11,720 | 1,137 | ATG/TAA |  | 6 |
| *trn-S2* | F | 11,726-11,793 | 68 |  | 11,755-11,757/TGA | 17 |
| *nad1* | R | 11,810-12,748 | 939 | ATA/TAG |  | 11 |
| *trn-L2* | R | 12,759-12,838 | 65 |  | 12,792-12,794/TAG | 1 |
| *rrnL-16S* | R | 14,161-12,824 | 1,338 |  |  | 1 |
| *trn-V* | R | 14,162-14,233 | 72 |  | 14,197-14,200/TAC | 1 |
| *rrnS-12S* | R | 14,234-15,036 | 803 |  |  | 1 |
| *D-loop* |  | 15,037-16,308 | 1,272 |  |  | 0 |
