# Supplementary material for: Eighteen mitochondrial genomes of Syrphidae (Insecta: Diptera: Brachycera) with a phylogenetic analysis of Muscomorpha
Source: PLoS One. 2023 Jan 5;18(1):e0278032. doi: 10.1371/journal.pone.0278032 (PMC9815649; doi:10.1371/journal.pone.0278032)
Supplement: S5 Table — (DOCX) [file pone.0278032.s064.docx]

**Supplementary Table 5** Gene organization of the complete mitogenome of *Epistrophe bashanensis*

| Gene | Direction | Location | Size (bp) | Start/stop codon | Anticodon | Intergennic nucleotide |
| --- | --- | --- | --- | --- | --- | --- |
| *trn-l* | F | 1-66 | 66 |  | 31-33/GAT |  |
| *trn-Q* | R | 92-160 | 69 |  | 128-130/TTG | 2 |
| *trn-M* | F | 167-234 | 69 |  | 197-199/CAT | 44 |
| *nad2* | F | 236-1,270 | 1,035 | ATT/TAA |  | 0 |
| *trn-W* | F | 1,281-1,348 | 68 |  | 1,312-1,314/TCA | -2 |
| *trn-C* | R | 1,353-1,419 | 67 |  | 1,388-1,390/GCA | 4 |
| *trn-Y* | R | 1,427-1,492 | 66 |  | 1,459-1,461/GTA | 7 |
| *cox1* | F | 1,493-3,070 | 1,578 | ATT/TAA |  | 0 |
| *trn-L1* | F | 3,066-3,131 | 66 |  | 3,095-3,097/TAA | -5 |
| *cox2* | F | 3,134-3,817 | 684 | ATG/TAA |  | 2 |
| *trn-K* | F | 3,818-3,888 | 71 |  | 3,848-3,850/CTT | 0 |
| *trn-D* | F | 3,998-3,931 | 68 |  | 3,963-3,965/GTC | 42 |
| *atp8* | F | 3,999-4,160 | 162 | ATT/TAA |  | 0 |
| *atp6* | F | 4,154-4,831 | 678 | ATG/TAA |  | -7 |
| *cox3* | F | 4,839-5,627 | 789 | ATG/TAA |  | 7 |
| *trn-G* | F | 5,631-5,696 | 66 |  | 5,660-5,662/TCC | 3 |
| *nad3* | F | 5,697-6,050 | 354 | ATT/TGA |  | 0 |
| *trn-A* | F | 6,049-6,117 | 69 |  | 6,082-6,084/TGC | -2 |
| *trn-R* | F | 6,117-6,180 | 64 |  | 6,146-6,148/TCG | -1 |
| *trn-N* | F | 6,207-6,273 | 67 |  | 6,238-6,240/GTT | 26 |
| *trn-S* | F | 6,274-6,340 | 67 |  | 6,299-6,301/GCT | 0 |
| *trn-E* | F | 6,346-6,411 | 66 |  | 6,375-6,377/TTC | 5 |
| *trn-F* | R | 6,432-6,497 | 66 |  | 6,463-6,465/GAA | 20 |
| *nad5* | R | 6,497-8,236 | 1,740 | ATT/TAA |  | -1 |
| *trn-H* | R | 8,234-8,299 | 66 |  | 8,267-8,269/GTG | -3 |
| *nad4* | R | 8,299-9,639 | 1,341 | ATG/TAA |  | -1 |
| *nad4L* | R | 9,633-9,929 | 297 | ATG/TAA |  | -7 |
| *trn-T* | F | 9,932-9,996 | 65 |  | 9,962-9,964/TGT | 0 |
| *trn-P* | R | 9,997-10,062 | 66 |  | 10,030-10,032/TGG | 2 |
| *nad6* | F | 10,065-10,589 | 525 | ATT/TAA |  | 3 |
| *cob* | F | 10,593-11,729 | 1,137 | ATG/TAA |  | 5 |
| *trn-S2* | F | 11,735-11,802 | 68 |  | 11,764-11,766/TGA | 16 |
| *nad1* | R | 11,819-12,748 | 939 | ATA/TAA |  | 11 |
| *trn-L2* | R | 12,769-12,832 | 64 |  | 12,801-12,803/TAG | 0 |
| *rrnL-16S* | R | 12,833-14,172 | 1,340 |  |  | 0 |
| *trn-V* | R | 14,173-14,244 | 72 |  | 14,209-14,211/TAC | 0 |
| *rrnS-12S* | R | 14,245-15,047 | 803 |  |  | 0 |
| *D-loop* |  | 15,048-16,272 | 1,225 |  |  | 0 |
