# Supplementary material for: Eighteen mitochondrial genomes of Syrphidae (Insecta: Diptera: Brachycera) with a phylogenetic analysis of Muscomorpha
Source: PLoS One. 2023 Jan 5;18(1):e0278032. doi: 10.1371/journal.pone.0278032 (PMC9815649; doi:10.1371/journal.pone.0278032)
Supplement: S6 Table — (DOCX) [file pone.0278032.s065.docx]

**Supplementary Table 6** Gene organization of the complete mitogenome of *Epistrophe lamellata*

| Gene | Direction | Location | Size (bp) | Start/stop codon | Anticodon | Intergennic nucleotide |
| --- | --- | --- | --- | --- | --- | --- |
| *trn-l* | F | 1-66 | 66 |  | 30-32/GAT | 0 |
| *trn-Q* | R | 64-132 | 69 |  | 104-106/GTT | -3 |
| *trn-M* | F | 138-206 | 69 |  | 168-170/CAT | 5 |
| *nad2* | F | 207-1,241 | 1,035 | ATA/TAA |  | 0 |
| *trn-W* | F | 1,240-1,308 | 69 |  | 1,271-1,273/TCA | -2 |
| *trn-C* | R | 1,325-1,390 | 66 |  | 1,359-1,361/TGC | 16 |
| *trn-Y* | R | 1,398-1,463 | 66 |  | 1,430-1,432/GTA | 7 |
| *cox1* | F | 1,469-3,040 | 1,572 | ATT/TAA |  | 5 |
| *trn-L* | F | 3,036-3,101 | 66 |  | 3,065-3,067/TAA | -5 |
| *cox2* | F | 3,103-3,789 | 687 | ATA/TAA |  | 1 |
| *trn-K* | F | 3,791-3,859 | 69 |  | 3,820-3,822/CTT | 1 |
| *trn-D* | F | 3,889-3,958 | 70 |  | 3,921-3,923/GTC | 29 |
| *atp8* | F | 3,956-4,120 | 165 | TTG/TAA |  | -3 |
| *atp6* | F | 4,108-4,791 | 684 | TTG/TAA |  | -13 |
| *cox3* | F | 4,799-5,587 | 789 | ATG/TAA |  | 7 |
| *trn-G* | F | 5,591-5,656 | 66 |  | 5,620-5,622/TCC | 3 |
| *nad3* | F | 5,654-6,010 | 357 | ATA/TAA |  | -3 |
| *trn-A* | F | 6,013-6,081 | 69 |  | 6,045-6,047/TGC | 2 |
| *trn-R* | F | 6,081-6,144 | 64 |  | 6,110-6,112/TCG | -1 |
| *trn-N* | F | 6,160-6,226 | 67 |  | 6,191-6,193/GTT | 15 |
| *trn-S1* | F | 6,227-6,293 | 67 |  | 6,252-6,254/GCT | 0 |
| *trn-E* | F | 6,295-6,362 | 68 |  | 6,325-6,327/TTC | 1 |
| *trn-F* | R | 6,381-6,448 | 68 |  | 6,412-6,414/GAA | 18 |
| *nad5* | R | 6,446-8,187 | 1,740 | ATC/TAA |  | -1 |
| *trn-H* | R | 8,185-8,250 | 66 |  | 8,214-8,216/GTG | -3 |
| *nad4* | R | 8,251-9,591 | 1,341 | ATG/TAA |  | 0 |
| *nad4L* | R | 9,585-9,881 | 297 | ATG/TAA |  | -7 |
| *trn-T* | F | 9,884-9,950 | 67 |  | 9,914-9,916/TGT | 2 |
| *trn-P* | R | 9,950-10,015 | 66 |  | 9,983-9,985/TGG | -1 |
| *nad6* | F | 10,018-10,542 | 525 | ATT/TAA |  | 2 |
| *cob* | F | 10,554-11,690 | 1,137 | ATG/TAA |  | 11 |
| *trn-S2* | F | 11,694-11,762 | 69 |  | 11,723-11,725/TGA | 3 |
| *nad1* | R | 11,779-12,717 | 939 | ATA/TAA |  | 16 |
| *trn-L2* | R | 12,728-12,792 | 65 |  | 12,761-12,763/TAG | 10 |
| *rrnL-16S* | R | 12,795-14,127 | 1,333 |  |  | 2 |
| *trn-V* | R | 14,200-14,129 | 72 |  | 14,165-14,167/TAC | 0 |
| *rrnS-12S* | R | 14,201-14,995 | 795 |  |  | 0 |
| *D-loop* |  | 14,996-16,405 | 1,410 |  |  | 0 |
