# Supplementary material for: Eighteen mitochondrial genomes of Syrphidae (Insecta: Diptera: Brachycera) with a phylogenetic analysis of Muscomorpha
Source: PLoS One. 2023 Jan 5;18(1):e0278032. doi: 10.1371/journal.pone.0278032 (PMC9815649; doi:10.1371/journal.pone.0278032)
Supplement: S7 Table — (DOCX) [file pone.0278032.s066.docx]

**Supplementary** **Table 7** Gene organization of the complete mitogenome of *Epistrophe latifasciatus*

| Gene | Direction | Location | Size (bp) | Start/stop codon | Anticodon | Intergennic nucleotide |
| --- | --- | --- | --- | --- | --- | --- |
| *trn-l* | F | 1-67 | 67 |  | 31-33/GAT |  |
| *trn-Q* | R | 108-176 | 69 |  | 144-146/TTG | 40 |
| *trn-M* | F | 184-252 | 69 |  | 214-216/CAT | 7 |
| *nad2* | F | 253-1,293 | 1,041 | ATT/TAA |  | 0 |
| *trn-W* | F | 1,301-1,368 | 68 |  | 1,332-1,334/TCA | 7 |
| *trn-C* | R | 1,372-1,438 | 67 |  | 1,407-1,409/GCA | 3 |
| *trn-Y* | R | 1,460-1,525 | 66 |  | 1,492-1,494/GTA | 21 |
| *cox1* | F | 1,534-3,081 | 1,548 | ATA/TAA |  | 9 |
| *trn-L1* | F | 3,077-3,142 | 66 |  | 3,106-3,108/TAA | -5 |
| *cox2* | F | 3,146-3,829 | 684 | ATG/TAA |  | 3 |
| *trn-K* | F | 3,830-3,900 | 71 |  | 3,860-3,862/CTT | 0 |
| *trn-D* | F | 3,939-4,005 | 67 |  | 3,970-3,972/GTC | 38 |
| *atp8* | F | 4,003-4,167 | 165 | TTG/TAA |  | 3 |
| *atp6* | F | 4,155-4,838 | 684 | TTG/TAA |  | -13 |
| *cox3* | F | 4,856-5,644 | 789 | ATG/TAA |  | 17 |
| *trn-G* | F | 5,648-5,713 | 66 |  | 5,677-5,679/TCC | 3 |
| *nad3* | F | 5,711-6,067 | 357 | ATA/TAA |  | -3 |
| *trn-A* | F | 6,074-6,140 | 67 |  | 6,105-6,107/TGC | 6 |
| *trn-R* | F | 6,140-6,203 | 64 |  | 6,169-6,171/TCG | -1 |
| *trn-N* | F | 6,218-6,284 | 67 |  | 6,249-6,251/GTT | 14 |
| *trn-S1* | F | 6,284-6,352 | 69 |  | 6,310-6,312/GCT | -1 |
| *trn-E* | F | 6,356-6,421 | 66 |  | 6,385-6,387/TTC | 3 |
| *trn-F* | R | 6,450-6,515 | 66 |  | 6,481-6,483/GAA | 28 |
| *nad5* | R | 6,515-8,254 | 1,740 | ATT/TAA |  | -1 |
| *trn-H* | R | 8,252-8,318 | 67 |  | 8,285-8,287/GTG | -3 |
| *nad4* | R | 8,321-9,661 | 1,341 | ATG/TAA |  | 2 |
| *nad4L* | R | 9,655-9,951 | 297 | ATG/TAA |  | -7 |
| *trn-T* | F | 9,954-10,018 | 65 |  | 9,984-9,986/TGT | 2 |
| *trn-P* | R | 10,019-10,084 | 66 |  | 10,052-10,054/TGG | 0 |
| *nad6* | F | 10,087-10,611 | 525 | ATC/TAA |  | 2 |
| *cob* | F | 10,619-11,755 | 1,137 | ATG/TAA |  | 7 |
| *trn-S2* | F | 11,759-11,826 | 68 |  | 11,788-11,790/TGA | 3 |
| *nad1* | R | 11,843-12,781 | 939 | ATA/TAA |  | 16 |
| *trn-L2* | R | 12,791-12,856 | 66 |  | 12,825-12,827/TAG | 9 |
| *rrnL-16S* | R | 12,857-14,201 | 1,345 |  |  | 0 |
| *trn-V* | R | 14,202-14,273 | 72 |  | 14,238-14,240/TAC | 0 |
| *rrnS-12S* | R | 14,274-15,068 | 795 |  |  | 0 |
| *D-loop* |  | 15,069-15,968 | 900 |  |  | 0 |
