# Supplementary material for: Eighteen mitochondrial genomes of Syrphidae (Insecta: Diptera: Brachycera) with a phylogenetic analysis of Muscomorpha
Source: PLoS One. 2023 Jan 5;18(1):e0278032. doi: 10.1371/journal.pone.0278032 (PMC9815649; doi:10.1371/journal.pone.0278032)
Supplement: S9 Table — (DOCX) [file pone.0278032.s068.docx]

**Supplementary Table 9** Gene organization of the complete mitogenome of *Eristalis arbustorum*

| Gene | Direction | Location | Size  (bp) | Anticode | start/stop codon | Intergenic Sequence |
| --- | --- | --- | --- | --- | --- | --- |
| *trn-l* | F | 1-66 | 66 | 30-32/GAT |  | 0 |
| *trn-Q* | R | 64-132 | 69 | 100-102/TTG |  | -3 |
| *trn-M* | F | 145-213 | 69 | 175-177/CAT |  | 12 |
| *nad2* | F | 214-1,236 | 1,023 |  | ATT/TAA | 0 |
| *trn-W* | F | 1,234-1,303 | 70 | 1,265-1,267/TCA |  | -3 |
| *trn-C* | R | 1,294-1,361 | 68 | 1,331-1,329/GCA |  | -10 |
| *trn-Y* | R | 1,365-1,431 | 67 | 1,398-1,400/GTA |  | 3 |
| *cox1* | F | 1,466-2,968 | 1,503 |  | ATT/TAA | 34 |
| *trn-L* | F | 2,963-3,030 | 68 | 2,993-2,995/TAA |  | -6 |
| *cox2* | F | 3,034-3,717 | 684 |  | ATG/TAA | 3 |
| *trn-K* | F | 3,719-3,789 | 71 | 3,749-3,751/CTT |  | 1 |
| *trn-D* | F | 3,842-3,909 | 68 | 3,873-3,875/GTC |  | 52 |
| *atp8* | F | 3,907-4,071 | 165 |  | TTG/TAA | -3 |
| *atp6* | F | 4,068-4,742 | 675 |  | ATA/TAA | -4 |
| *cox3* | F | 4,755-5,543 | 789 |  | ATG/TAA | 12 |
| *trn-G* | F | 5,547-5,613 | 67 | 5,576-5,578/TCC |  | 3 |
| *nad3* | F | 5,611-5,967 | 357 |  | ATA/TAA | -3 |
| *trn-A* | F | 5972-6039 | 68 | 6,003-6,005/TGC |  | 4 |
| *trn-R* | F | 6,039-6,101 | 63 | 6,068-6,070/TCG |  | -1 |
| *trn-N* | F | 6,111-6,176 | 66 | 6,142-6,144/GTT |  | 9 |
| *trn-S1* | F | 6,177-6,243 | 67 | 6,202-6,204/GCT |  | 0 |
| *trn-E* | F | 6,244-6,309 | 66 | 6,274-6,276/TTC |  | 0 |
| *trn-F* | R | 6,348-6,414 | 67 | 6,380-6,382/GAA |  | 38 |
| *nad5* | R | 6,415-8,149 | 1,735 |  | ATT/T-- | 0 |
| *trn-H* | R | 8,147-8,212 | 66 | 8,180-8,182/GTG |  | -3 |
| *nad4* | R | 8,212-9,552 | 1,341 |  | ATG/TAA | -1 |
| *nad4L* | R | 9,546-9,842 | 297 |  | ATG/TAA | -7 |
| *trn-T* | F | 9,844-9,911 | 68 | 9,875-9,877/TGT |  | 1 |
| *trn-P* | R | 9,911-9,976 | 66 | 9,944-9,946/TGG |  | -1 |
| *nad6* | F | 9,979-10,503 | 525 |  | ATT/TAA | 2 |
| *cob* | F | 10,503-11,639 | 1,137 |  | ATT/TAA | -1 |
| *trn-S2* | F | 11,642-11,709 | 68 | 11,684-11,686/TAG |  | 2 |
| *nad1* | R | 11,732-12,673 | 942 |  | TTG/TAA | 22 |
| *trn-L2* | R | 12,675-12,739 | 65 | 12,708-12,710/TAG |  | 1 |
| *rrnL-16S* | R | 12,740-14,074 | 1,335 |  |  | 0 |
| *trn-V* | R | 14,075-14,146 | 72 | 14,111-14,113/TAC |  | 0 |
| *rrnS-12S* | R | 14,147-14,934 | 788 |  |  | 0 |
| *D-loop* |  | 14,935-15,983 | 1,049 |  |  | 0 |
