# Supplementary material for: Eighteen mitochondrial genomes of Syrphidae (Insecta: Diptera: Brachycera) with a phylogenetic analysis of Muscomorpha
Source: PLoS One. 2023 Jan 5;18(1):e0278032. doi: 10.1371/journal.pone.0278032 (PMC9815649; doi:10.1371/journal.pone.0278032)
Supplement: S10 Table — (DOCX) [file pone.0278032.s069.docx]

**Supplementary Table 10** Gene organization of the complete mitogenome of *Eristalis himalayensis*

| Gene | Direction | Location | Size (bp) | Start/stop codon | Anticodon | Intergennic nucleotide |
| --- | --- | --- | --- | --- | --- | --- |
| *trn-l* | F | 1-66 | 66 |  | 30-32/GAT | 0 |
| *trn-Q* | R | 64-132 | 69 |  | 102-100/TTG | -3 |
| *trn-M* | F | 140-208 | 69 |  | 170-172/CAT | 7 |
| *nad2* | F | 209-1,231 | 1,023 | ATT/TAA |  | 0 |
| *trn-W* | F | 1,230-1,298 | 69 |  | 1,260-1,262/TCA | -2 |
| *trn-C* | R | 1291-1,358 | 68 |  | 1,329-1,327/GCA | -8 |
| *trn-Y* | R | 1,362-1,428 | 67 |  | 1,397-1,395/GTA | 3 |
| *cox1* | F | 1,463-2,965 | 1,503 | ATT/TAA |  | 34 |
| *trn-L1* | F | 2,961-3,026 | 66 |  | 2,990-2,992/TAA | -5 |
| *cox2* | F | 3,026-3,718 | 693 | ATA/TAA |  | -1 |
| *trn-K* | F | 3,720-3,790 | 71 |  | 3,750-3,752/CTT | 1 |
| *trn-D* | F | 3,795-3,860 | 66 |  | 3,824-3,826/GTC | 4 |
| *atp8* | F | 3,858-4,022 | 165 | TTG/TAA |  | -3 |
| *atp6* | F | 4,019-4,693 | 675 | ATA/TAA |  | -4 |
| *cox3* | F | 4,703-5,491 | 789 | ATG/TAA |  | 9 |
| *trn-G* | F | 5,495-5,561 | 67 |  | 5,524-5,526/TCC | 3 |
| *nad3* | F | 5,562-5,915 | 354 | ATT/TAG |  | 0 |
| *trn-A* | F | 5,914-5,980 | 67 |  | 5,944-5,946/TGC | -2 |
| *trn-R* | F | 5,980-6,042 | 63 |  | 6,009-6,011/TCG | -1 |
| *trn-N* | F | 6,057-6,122 | 66 |  | 6,088-6,090/GTT | 14 |
| *trn-S* | F | 6,123-6,189 | 67 |  | 6,148-6,150/GCT | 0 |
| *trn-E* | F | 6,190-6,255 | 66 |  | 6,220-6,222/TTC | 0 |
| *trn-F* | R | 6,289-6,355 | 67 |  | 6,323-6,321/GAA | 32 |
| *nad5* | R | 6,356-8,090 | 1,735 | ATT/T-- |  | 0 |
| *trn-H* | R | 8,088-8,153 | 66 |  | 8,123-8,121/GTG | -3 |
| *nad4* | R | 8,154-9,494 | 1,341 | ATG/TAA |  | 0 |
| *nad4L* | R | 9,488-9,784 | 297 | ATG/TAA |  | -7 |
| *trn-T* | F | 9,787-9,852 | 66 |  | 9,817-9,819/TGT | 2 |
| *trn-P* | R | 9,853-9,918 | 66 |  | 9,888-9,886/TGG | 0 |
| *nad6* | F | 9,921-10,445 | 525 | ATT/TAA |  | 2 |
| *cob* | F | 10,445-11,581 | 1,137 | ATG/TAA |  | -1 |
| *trn-S2* | F | 11,584-11,651 | 68 |  | 11,613-11,615/TGA | 2 |
| *nad1* | R | 11,673-12,614 | 942 | TTG/TAG |  | 21 |
| *trn-L2* | R | 12,616-12,680 | 65 |  | 12,651-12,649/TAG | 1 |
| *rrnL-16S* | R | 12,681-14,018 | 1,338 |  |  | 0 |
| *trn-V* | R | 14,019-14,090 | 72 |  | 14,057-14,055/TAC | 0 |
| *rrnS-12S* | R | 14,091-14,880 | 790 |  |  | 0 |
| *D-loop* |  | 14,881-16,008 | 1,128 |  |  | 0 |
