# Supplementary material for: Eighteen mitochondrial genomes of Syrphidae (Insecta: Diptera: Brachycera) with a phylogenetic analysis of Muscomorpha
Source: PLoS One. 2023 Jan 5;18(1):e0278032. doi: 10.1371/journal.pone.0278032 (PMC9815649; doi:10.1371/journal.pone.0278032)
Supplement: S11 Table — (DOCX) [file pone.0278032.s070.docx]

**Supplementary** **Table 11** Gene organization of the complete mitogenome of *Eupeodes confrater*

| Gene | Direction | Location | Size (bp) | Start/stop codon | Anticodon | Intergennic nucleotide |
| --- | --- | --- | --- | --- | --- | --- |
| *trn-l* | F | 1-66 | 66 |  | 30-32/GAT |  |
| *trn-Q* | R | 64-132 | 69 |  | 100-102/GTT | -3 |
| *trn-M* | F | 144-212 | 69 |  | 174-176/CAT | 11 |
| *nad2* | F | 213-1,241 | 1,029 | ATT/TAA |  | 0 |
| *trn-W* | F | 1,240-1,307 | 68 |  | 1,271-1,273/TCA | -2 |
| *trn-C* | R | 1,314-1,383 | 70 |  | 1,352-1,354/GCA | 6 |
| *trn-Y* | R | 1,399-1,464 | 66 |  | 1,431-1,433/GTA | 15 |
| *cox1* | F | 1,467-3,071 | 1,614 | ATT/TAA |  | 2 |
| *trn-L* | F | 3,013-3,078 | 66 |  | 3,042-3,044/TAA | -5 |
| *cox2* | F | 3,081-3,764 | 684 | ATG/TAA |  | 2 |
| *trn-K* | F | 3,765-3,835 | 71 |  | 3,795-3,797/CTT | 0 |
| *trn-D* | F | 3,872-3,938 | 67 |  | 3,903-3,905/GTC | 36 |
| *atp8* | F | 3,939-4,100 | 162 | ATT/TAA |  | -7 |
| *atp6* | F | 4,094-4,771 | 678 | ATG/TAA |  | -13 |
| *cox3* | F | 4,789-5,577 | 789 | ATG/TAA |  | 17 |
| *trn-G* | F | 5,581-5,647 | 67 |  | 5,610-5,612TCC | 3 |
| *nad3* | F | 5,648-6,001 | 354 | ATT/TAA |  | 0 |
| *trn-A* | F | 6,005-6,073 | 69 |  | 6,036-6,038/TGC | 3 |
| *trn-R* | F | 6,073-6,136 | 64 |  | 6,102-6,104/TCG | -1 |
| *trn-N* | F | 6,151-6,217 | 67 |  | 6,182-6,184/GTT | 14 |
| *trn-S* | F | 6,217-6,285 | 69 |  | 6,243-6,245/GCT | -1 |
| *trn-E* | F | 6,289-6,356 | 68 |  | 6,321-6,323/TTC | 3 |
| *trn-F* | R | 6,384-6,450 | 67 |  | 6,415-6,417/GAA | 27 |
| *nad5* | R | 6,434-8,188 | 1,755 | ATT/TAA |  | 17 |
| *trn-H* | R | 8,186-8,251 | 66 |  | 8,219-8,221/GTG | -3 |
| *nad4* | R | 8,251-9,591 | 1,341 | ATG/TAA |  | -1 |
| *nad4L* | R | 9,585-9,881 | 297 | ATG/TAA |  | -7 |
| *trn-T* | F | 9,884-9,948 | 65 |  | 9,914-9,916/TGT | 2 |
| *trn-P* | R | 9,949-10,014 | 66 |  | 9,982-9,984/TGG | 0 |
| *nad6* | F | 10,017-10,541 | 525 | ATT/TAA |  | 2 |
| *cob* | F | 10,545-11,681 | 1,137 | ATG/TAA |  | 3 |
| *trn-S2* | F | 11,689-11,756 | 68 |  | 1,1718-1,1720/TGA | 7 |
| *nad1* | R | 11,773-12,711 | 939 | ATA/TAG |  | 16 |
| *trn-L2* | R | 12,722-12,786 | 65 |  | 1,2755-1,2757/TAG | 10 |
| *rrnL-16S* | R | 12,787-14,124 | 1,338 |  |  | 0 |
| *trn-V* | R | 14,125-14,196 | 72 |  | 14,161-14,163/TAC | 0 |
| *rrnS-12S* | R | 14,197-14,992 | 796 |  |  | 0 |
| *D-loop* |  | 14,993-16,175 | 1,183 |  |  | 0 |
