# Supplementary material for: Eighteen mitochondrial genomes of Syrphidae (Insecta: Diptera: Brachycera) with a phylogenetic analysis of Muscomorpha
Source: PLoS One. 2023 Jan 5;18(1):e0278032. doi: 10.1371/journal.pone.0278032 (PMC9815649; doi:10.1371/journal.pone.0278032)
Supplement: S12 Table — (DOCX) [file pone.0278032.s071.docx]

**Supplementary Table 12** Gene organization of the complete mitogenome of *Eupeodes luniger*

| Gene | Direction | Location | Size (bp) | Start/stop codon | Anticodon | Intergennic nucleotide |
| --- | --- | --- | --- | --- | --- | --- |
| *trn-l* | F | 1-66 | 66 |  | 30-32/GAT | -1 |
| *trn-Q* | R | 65-131 | 67 |  | 100-102/TTG | 9 |
| *trn-M* | F | 140-208 | 69 |  | 170-172/CAT | 1 |
| *nad2* | F | 209-1,237 | 1,029 | ATT/TAT |  | -1 |
| *trn-W* | F | 1,236-1,303 | 68 |  | 1,267-1,269/TCA | 5 |
| *trn-C* | R | 1,308-1,373 | 66 |  | 1,342-1,344/GCA | 10 |
| *trn-Y* | R | 1,383-1,448 | 66 |  | 1,415-1,417/GTA | 18 |
| *cox1* | F | 1,466-3,028 | 1,563 | ATT/TAA |  | -4 |
| *trn-L* | F | 3,024-3,090 | 67 |  | 3,053-3,055/TAA | 2 |
| *cox2* | F | 3,092-3,775 | 684 | ATG/TAA |  | 1 |
| *trn-K* | F | 3,776-3,846 | 71 |  | 3,806-3,808/CTT | 30 |
| *trn-D* | F | 3,876-3,942 | 67 |  | 3,907-3,909/GTC | 1 |
| *atp8* | F | 3,943-4,104 | 162 | ATC/TAA |  | -6 |
| *atp6* | F | 4,098-4,775 | 678 | ATG/TAA |  | 17 |
| *cox3* | F | 4,792-5,580 | 789 | ATG/TAA |  | 4 |
| *trn-G* | F | 5,584-5,650 | 67 |  | 5,613-5,615/TCC | 1 |
| *nad3* | F | 5,651-6,004 | 354 | ATT/TAA |  | 4 |
| *trn-A* | F | 6,008-6,076 | 69 |  | 6,039-6,041/TGC | 0 |
| *trn-R* | F | 6,076-6,139 | 64 |  | 6,105-6,107/TCG | 14 |
| *trn-N* | F | 6,153-6,219 | 67 |  | 6,184-6,187/GTT | 0 |
| *trn-S* | F | 6,129-6,287 | 69 |  | 6,245-6,247/GCT | 4 |
| *trn-E* | F | 6,291-6,357 | 67 |  | 6,321-6,323/TTC | 27 |
| *trn-F* | R | 6,384-6,450 | 67 |  | 6,415-6,417/GAA | -16 |
| *nad5* | R | 6,434-8,188 | 1,755 | ATT/TAA |  | -2 |
| *trn-H* | R | 8,186-8,251 | 66 |  | 8,219-8,221/GTG | 0 |
| *nad4* | R | 8,251-9,591 | 1,341 | ATG/TAA |  | 0 |
| *nad4L* | R | 9,585-9,881 | 297 | ATG/TAA |  | 3 |
| *trn-T* | F | 9,884-9,948 | 65 |  | 9,914-9,916/TGT | 1 |
| *trn-P* | R | 9,949-10,014 | 66 |  | 9,982-9,984/TGG | 3 |
| *nad6* | F | 10,017-10,541 | 525 | ATT/TAA |  | 4 |
| *cob* | F | 10,545-11,681 | 1,137 | ATG/TAA |  | 8 |
| *trn-S2* | F | 11,689-11,756 | 68 |  | 11,718-11,721/TGA | 17 |
| *nad1* | R | 11,773-12,711 | 939 | ATA/TAG |  | 10 |
| *trn-L2* | R | 12,721-12,786 | 66 |  | 12,755-12,757/TAG | 1 |
| *rrnL-16S* | R | 12,787-14,122 | 1,336 |  |  | 1 |
| *trn-V* | R | 14,123-14,194 | 72 |  | 14,159-14,161/TAC | 1 |
| *rrnS-12S* | R | 14,195-14,987 | 793 |  |  | 0 |
| *D-loop* |  | 14,988-16,182 | 1.195 |  |  | 0 |
