# Supplementary material for: Eighteen mitochondrial genomes of Syrphidae (Insecta: Diptera: Brachycera) with a phylogenetic analysis of Muscomorpha
Source: PLoS One. 2023 Jan 5;18(1):e0278032. doi: 10.1371/journal.pone.0278032 (PMC9815649; doi:10.1371/journal.pone.0278032)
Supplement: S13 Table — (DOCX) [file pone.0278032.s072.docx]

**Supplementary Table 13** Gene organization of the complete mitogenome of *Mallota bellus*

| Gene | Direction | Location | Size (bp) | Start/stop codon | Anticodon | Intergennic nucleotide |
| --- | --- | --- | --- | --- | --- | --- |
| *trn-l* | F | 1-66 | 66 |  | 30-32/GAT | 0 |
| *trn-Q* | R | 64-132 | 69 |  | 102-100/TTG | -3 |
| *trn-M* | F | 137-205 | 69 |  | 167-169/CAT | 4 |
| *nad2* | F | 206-1,228 | 1,023 | ATT/TAA |  | 0 |
| *trn-W* | F | 1,226-1,295 | 70 |  | 1,257-1,259/TCA | 3 |
| *trn-C* | R | 1,287-1,353 | 67 |  | 1,324-1,322/GCA | -9 |
| *trn-Y* | R | 1,356-1,422 | 67 |  | 1,391-1,389/GTA | 2 |
| *cox1* | F | 1,457-2,959 | 1,503 | ATT/TAA |  | 34 |
| *trn-L1* | F | 2,955-3,019 | 65 |  | 2,984-2,986/TAA | -5 |
| *cox2* | F | 3,023-3,706 | 684 | ATG/TAA |  | 3 |
| *trn-K* | F | 3,708-3,778 | 71 |  | 3,738-3,740/CTT | 1 |
| *trn-D* | F | 3,778-3,844 | 67 |  | 3,809-3,811/GTC | -1 |
| *atp8* | F | 3,845-4,006 | 162 | ATC/TAA |  | 0 |
| *atp6* | F | 4,003-4,677 | 675 | ATA/TAA |  | -4 |
| *cox3* | F | 4,683-5,471 | 789 | ATG/TAA |  | 5 |
| *trn-G* | F | 5,475-5,540 | 66 |  | 5,504-5,506/TCC | 3 |
| *nad3* | F | 5,538-5,894 | 357 | ATA/TAG |  | -3 |
| *trn-A* | F | 5,893-5,960 | 68 |  | 5,924-5,926/TGC | -2 |
| *trn-R* | F | 5,960-6,023 | 64 |  | 5,989-5,991/TCG | -1 |
| *trn-N* | F | 6,027-6,092 | 66 |  | 6,058-6,060/GTT | 3 |
| *trn-S* | F | 6,093-6,159 | 67 |  | 6,118-6,120/GCT | 0 |
| *trn-E* | F | 6,160-6,225 | 66 |  | 6,190-6,192/TTC | 0 |
| *trn-F* | R | 6,255-6,321 | 67 |  | 6,289-6,287/GAA | 29 |
| *nad5* | R | 6,322-8,056 | 1,735 | ATT/T-- |  | 0 |
| *trn-H* | R | 8,054-8,119 | 66 |  | 8,087-8,089/GTG | -3 |
| *nad4* | R | 8,125-9,465 | 1,341 | ATG/TAA |  | 5 |
| *nad4L* | R | 9,459-9,755 | 297 | ATG/TAA |  | -7 |
| *trn-T* | F | 9,758-9,823 | 66 |  | 9,788-9,790/TGT | 2 |
| *trn-P* | R | 9,824-9,889 | 66 |  | 9,859-9,857/TGG | 0 |
| *nad6* | F | 9,892-10,416 | 525 | ATT/TAA |  | 2 |
| *cob* | F | 10,416-11,552 | 1,137 | ATG/TAA |  | -1 |
| *trn-S2* | F | 11,558-11,625 | 68 |  | 11,587-11,589/TGA | 5 |
| *nad1* | R | 11,648-12,589 | 942 | TTG/TAA |  | 22 |
| *trn-L2* | R | 12,591-12,655 | 65 |  | 12,626-12,624/TAG | 1 |
| *rrnL-16S* | R | 12,656-13,985 | 1,330 |  |  | 0 |
| *trn-V* | R | 13,986-14,057 | 72 |  | 14,024-14,022/TAC | 0 |
| *rrnS-12S* | R | 14,058-14,851 | 794 |  |  | 0 |
| *D-loop* |  | 14,852-15,763 | 912 |  |  | 0 |
