# Supplementary material for: Eighteen mitochondrial genomes of Syrphidae (Insecta: Diptera: Brachycera) with a phylogenetic analysis of Muscomorpha
Source: PLoS One. 2023 Jan 5;18(1):e0278032. doi: 10.1371/journal.pone.0278032 (PMC9815649; doi:10.1371/journal.pone.0278032)
Supplement: S14 Table — (DOCX) [file pone.0278032.s073.docx]

**Supplementary Table 14** Gene organization of the complete mitogenome of *Mallota vilis*

| Gene | Direction | Location | Size (bp) | Start/stop codon | Anticodon | Intergennic nucleotide |
| --- | --- | --- | --- | --- | --- | --- |
| *trn-l* | F | 1-66 | 66 |  | 30-32/GAT | 0 |
| *trn-Q* | R | 70-138 | 69 |  | 108-106/TTG | 3 |
| *trn-M* | F | 138-206 | 69 |  | 168-170/CAT | -1 |
| *nad2* | F | 207-1,229 | 1,023 | ATT/TAA |  | 0 |
| *trn-W* | F | 1,227-1,296 | 70 |  | 1,258-1,260/TCA | -3 |
| *trn-C* | R | 1,288-1,353 | 66 |  | 1,324-1,322/GCA | -9 |
| *trn-Y* | R | 1,356-1,422 | 67 |  | 1,391-1,389/GTA | 2 |
| *cox1* | F | 1,457-2,959 | 1,503 | ATT/TAA |  | 34 |
| *trn-L1* | F | 2,954-3,021 | 68 |  | 2,984-2,986/TAA | -6 |
| *cox2* | F | 3,027-3,710 | 684 | ATG/TAA |  | 5 |
| *trn-K* | F | 3,712-3,782 | 71 |  | 3,742-3,744/CTT | 1 |
| *trn-D* | F | 3,811-3,877 | 67 |  | 3,842-3,844/GTC | 28 |
| *atp8* | F | 3,875-4,036 | 162 | TTG/TAA |  | -3 |
| *atp6* | F | 4,024-4,707 | 684 | TTG/TAA |  | -13 |
| *cox3* | F | 4,708-5,496 | 789 | ATG/TAA |  | 0 |
| *trn-G* | F | 5,500-5,565 | 66 |  | 5,529-5,531/TCC | 3 |
| *nad3* | F | 5,563-5,919 | 357 | ATG/TAG |  | -3 |
| *trn-A* | F | 5,918-5,985 | 68 |  | 5,949-5,951/TGC | -10 |
| *trn-R* | F | 5,985-6,046 | 62 |  | 6,014-6,016/TCG | -1 |
| *trn-N* | F | 6,049-6,114 | 66 |  | 6,080-6,082/GTT | 2 |
| *trn-S* | F | 6,114-6,182 | 69 |  | 6,140-6,142/GCT | -1 |
| *trn-E* | F | 6,182-6,247 | 66 |  | 6,212-6,214/TTC | -1 |
| *trn-F* | R | 6,271-6,336 | 66 |  | 6,304-6,302/GAA | 23 |
| *nad5* | R | 6,337-8,071 | 1,735 | ATT/T-- |  | 0 |
| *trn-H* | R | 8,069-8,134 | 66 |  | 8,104-8,102/GTG | -3 |
| *nad4* | R | 8,139-9,479 | 1,341 | ATG/TAA |  | 4 |
| *nad4L* | R | 9,473-9,769 | 297 | ATG/TAA |  | -7 |
| *trn-T* | F | 9,772-9,837 | 66 |  | 9,802-9,804/TGT | 2 |
| *trn-P* | R | 9,838-9,904 | 67 |  | 9,873-9,871/TGG | 0 |
| *nad6* | F | 9,907-10,431 | 525 | ATC/TAA |  | 2 |
| *cob* | F | 10,431-11,567 | 1,137 | ATG/TAA |  | -1 |
| *trn-S2* | F | 11,570-11,638 | 69 |  | 11,600-11,602/TGA | 2 |
| *nad1* | R | 11,660-12,601 | 942 | TTG/TAA |  | 21 |
| *trn-L2* | R | 12,603-12,667 | 65 |  | 12,638-12,636/TAG | 1 |
| *rrnL-16S* | R | 12,668-14,003 | 1,336 |  |  | 0 |
| *trn-V* | R | 14,004-14,075 | 72 |  | 14,042-14,040/TAC | 0 |
| *rrnS-12S* | R | 14,076-14,864 | 789 |  |  | 0 |
| *D-loop* | F | 14,865-15,802 | 938 |  |  | 0 |
