# Supplementary material for: Eighteen mitochondrial genomes of Syrphidae (Insecta: Diptera: Brachycera) with a phylogenetic analysis of Muscomorpha
Source: PLoS One. 2023 Jan 5;18(1):e0278032. doi: 10.1371/journal.pone.0278032 (PMC9815649; doi:10.1371/journal.pone.0278032)
Supplement: S15 Table — (DOCX) [file pone.0278032.s074.docx]

**Supplementary Table 15** Gene organization of the complete mitogenome of *Mesembrius niger*

| Gene | Direction | Location | Size (bp) | Start/stop codon | Anticodon | Intergennic nucleotide |
| --- | --- | --- | --- | --- | --- | --- |
| *trn-l* | F | 1-66 | 66 |  | 21-23/GAT | 0 |
| *trn-Q* | R | 64-132 | 69 |  | 102-100/TTG | -3 |
| *trn-M* | F | 156-224 | 69 |  | 186-188/CAT | 23 |
| *nad2* | F | 225-1,247 | 1,023 | ATT/TAA |  | 0 |
| *trn-W* | F | 1,245-1,314 | 70 |  | 1,276-1,278/TCA | -2 |
| *trn-C* | R | 1,332-1,397 | 66 |  | 1,368-1,366/GCA | 17 |
| *trn-Y* | R | 1,402-1,468 | 67 |  | 1,437-1,435/GTA | 4 |
| *cox1* | F | 1,507-3,009 | 1,503 | ATT/TAA |  | 38 |
| *trn-L1* | F | 3,005-3,070 | 66 |  | 3,034-3,036/TAA | -5 |
| *cox2* | F | 3,072-3,758 | 687 | ATA/TAA |  | 1 |
| *trn-K* | F | 3,760-3,830 | 71 |  | 3,790-3,792/CTT | 1 |
| *trn-D* | F | 3,844-3,911 | 68 |  | 3,876-3,878/GTC | 13 |
| *atp8* | F | 3,912-4,073 | 162 | ATT/TAA |  | 0 |
| *atp6* | F | 4,070-4,744 | 675 | ATA/TAA |  | -4 |
| *cox3* | F | 4,750-5,538 | 789 | ATG/TAA |  | 5 |
| *trn-G* | F | 5,542-5,610 | 69 |  | 5,571-5,573/TCC | 3 |
| *nad3* | F | 5563-5,919 | 357 | ATA/TAG |  | -3 |
| *trn-A* | F | 5976-6,041 | 66 |  | 6,006-6,008/TGC | 11 |
| *trn-R* | F | 6041-6,103 | 63 |  | 6,070-6,072/TCG | -1 |
| *trn-N* | F | 6107-6,172 | 66 |  | 6,138-6,140/GTT | 3 |
| *trn-S* | F | 6173-6,239 | 67 |  | 6,195-6,197/GCT | 0 |
| *trn-E* | F | 6,240-6,308 | 69 |  | 6,272-6,274/TTC | 0 |
| *trn-F* | R | 6,331-6,398 | 68 |  | 6,365-6,363/GAA | 22 |
| *nad5* | R | 6,398-8,134 | 1,737 | ATT/TAA |  | -1 |
| *trn-H* | R | 8,132-8,197 | 66 |  | 8,167-8,165/GTG | -3 |
| *nad4* | R | 8,198-9,538 | 1,341 | ATG/TAA |  | 0 |
| *nad4L* | R | 9,532-9,822 | 291 | ATA/TAA |  | -7 |
| *trn-T* | F | 9,831-9,896 | 66 |  | 9,861-9,863/TGT | 8 |
| *trn-P* | R | 9,896-9,963 | 68 |  | 9,932-9,930/TGG | -1 |
| *nad6* | F | 9,965-10,489 | 525 | ATC/TAA |  | 1 |
| *cob* | F | 10,489-11,625 | 1,137 | ATG/TAA |  | -1 |
| *trn-S2* | F | 11,625-11,692 | 68 |  | 11,654-11,656/TGA | -1 |
| *nad1* | R | 11,714-12,655 | 942 | TTG/TAA |  | 21 |
| *trn-L2* | R | 12,657-12,721 | 65 |  | 12,692-12,690/TAG | -1 |
| *rrnL-16S* | R | 12,722-14,058 | 1,337 |  |  | 0 |
| *trn-V* | R | 14,059-14,130 | 72 |  | 14,097-14,095/TAC | 0 |
| *rrnS-12S* | R | 14,131-14,934 | 804 |  |  | 0 |
| *D-loop* | F | 14,935-15,824 | 890 |  |  | 0 |
