# Supplementary material for: Eighteen mitochondrial genomes of Syrphidae (Insecta: Diptera: Brachycera) with a phylogenetic analysis of Muscomorpha
Source: PLoS One. 2023 Jan 5;18(1):e0278032. doi: 10.1371/journal.pone.0278032 (PMC9815649; doi:10.1371/journal.pone.0278032)
Supplement: S16 Table — (DOCX) [file pone.0278032.s075.docx]

**Supplementary Table 16** Gene organization of the complete mitogenome of *Mallota viridiflavescentis*

| Gene | Direction | Location | Size (bp) | start/stop codon | Anticodon | Intergenic Sequence |
| --- | --- | --- | --- | --- | --- | --- |
| *trn-l* | F | 1-65 | 65 |  | 30-32 GAT | 0 |
| *trn-Q* | R | 64-130 | 67 |  | 101-99 TTG | -2 |
| *trn-M* | F | 154-222 | 69 |  | 184-186 CAT | 23 |
| *nad2* | F | 223-1245 | 1023 | ATT/TAA |  | 0 |
| *trn-W* | F | 1243-1312 | 70 |  | 1274-1276 TCA | -3 |
| *trn-C* | R | 1304-1369 | 66 |  | 1340-1338 GCA | -9 |
| *trn-Y* | R | 1376-1442 | 67 |  | 1411-1409 GTA | 6 |
| *cox1* | F | 1477-2979 | 1503 | ATT/TAA |  | 34 |
| *trn-L* | F | 2975-3040 | 66 |  | 3004-3006 TAA | -5 |
| *cox2* | F | 3049-3732 | 684 | ATG/TAA |  | 8 |
| *trn-K* | F | 3734-3804 | 71 |  | 3764-3766 CTT | 1 |
| *trn-D* | F | 3828-3904 | 77 |  | 3859-3861 GTC | 23 |
| *atp8* | F | 3895-4056 | 162 | ATT/TAA |  | -10 |
| *atp6* | F | 4053-4727 | 675 | ATA/TAA |  | -4 |
| *cox3* | F | 4734-5522 | 789 | ATG/TAA |  | 6 |
| *trn-G* | F | 5526-5591 | 66 |  | 5555-5557 TCC | 3 |
| *nad3* | F | 5589-5945 | 357 | ATA/TAA |  | -3 |
| *trn-A* | F | 5948-6015 | 68 |  | 5979-5981 TGC | 2 |
| *trn-R* | F | 6015-6077 | 63 |  | 6044-6046 TCG | -1 |
| *trn-N* | F | 6082-6147 | 66 |  | 6113-6115 GTT | 4 |
| *trn-S1* | F | 6148-6215 | 68 |  | 6174-6176 GCT | 0 |
| *trn-E* | F | 6216-6281 | 66 |  | 6246-6248 TTC | 0 |
| *trn-F* | R | 6310-6375 | 66 |  | 6343-6341 GAA | 28 |
| *nad5* | R | 6376-8110 | 1735 | ATT/T-- |  | 0 |
| *trn-H* | R | 8108-8173 | 66 |  | 8143-8141 GTG | -3 |
| *nad4* | R | 8174-9514 | 1341 | ATG/TAA |  | 0 |
| *nad4L* | R | 9508-9804 | 297 | ATG/TAA |  | -7 |
| *trn-T* | F | 9807-9872 | 66 |  | 9837-9839 TGT | 2 |
| *trn-P* | R | 9873-9938 | 66 |  | 9906-9908 TGG | 0 |
| *nad6* | F | 9941-10465 | 525 | ATC/TAA |  | 2 |
| *cob* | F | 10465-11601 | 1137 | ATG/TAA |  | -1 |
| *trn-S2* | F | 11604-11671 | 68 |  | 11633-11635 TGA | 2 |
| *nad1* | R | 11693-12634 | 942 | TTG/TAA |  | 21 |
| *trn-L2* | R | 12636-12700 | 65 |  | 12671-12669 TAG | 1 |
| *rrnL-16S* | R | 12701-14037 | 1337 |  |  | 0 |
| *trn-V* | R | 14038-14109 | 72 |  | 14076-14074 TAC | 0 |
| *rrnS-12S* | R | 14110-14910 | 801 |  |  | 0 |
| *D-loop* | F | 14911-15911 | 1001 |  |  | 0 |
