# Supplementary material for: Eighteen mitochondrial genomes of Syrphidae (Insecta: Diptera: Brachycera) with a phylogenetic analysis of Muscomorpha
Source: PLoS One. 2023 Jan 5;18(1):e0278032. doi: 10.1371/journal.pone.0278032 (PMC9815649; doi:10.1371/journal.pone.0278032)
Supplement: S17 Table — (DOCX) [file pone.0278032.s076.docx]

**Supplementary Table 17** Gene organization of the complete mitogenome of *Parhelophilus kurentzovi*

| Gene | Direction | Location | Size  (bp) | Start/stop codon | Anticodon | Intergenic Sequence |
| --- | --- | --- | --- | --- | --- | --- |
| *trn-l* | F | 1-66 | 66 |  | 30-32/GAT | 0 |
| *trn-Q* | R | 64-132 | 69 |  | 102-100/TTG | -3 |
| *trn-M* | F | 147-215 | 69 |  | 177-179/CAT | 15 |
| *nad2* | F | 216-1,235 | 1,020 | ATT/TAA |  | 0 |
| *trn-W* | F | 1,235-1,302 | 68 |  | 1,265-1,267/TCA | -1 |
| *trn-C* | R | 1,295-1,360 | 66 |  | 1,331-1,329/GCA | -7 |
| *trn-Y* | R | 1,362-1,427 | 66 |  | 1,396-1,394/ GTA | 2 |
| *cox1* | F | 1,462-2,964 | 1,503 | ATT/TAA |  | 34 |
| *trn-L* | F | 2,960-3,025 | 66 |  | 2,989-2,991/TAA | -5 |
| *cox2* | F | 3,029-3,712 | 684 | ATG/TAA |  | 3 |
| *trn-K* | F | 3,714-3,784 | 71 |  | 3,744-3,746/CTT | 1 |
| *trn-D* | F | 3,787-3,852 | 66 |  | 3,817-3,819/GTC | 2 |
| *atp8* | F | 3,853-4,014 | 162 | ATC/TAA |  | 0 |
| *atp6* | F | 4,011-,4685 | 675 | ATA/TAA |  | -4 |
| *cox3* | F | 4,685-5,473 | 789 | ATG/TAA |  | -1 |
| *trn-G* | F | 5,478-5,544 | 67 |  | 5,507-5,509/TCC | 4 |
| *nad3* | F | 5,542-5,898 | 357 | ATA/TAA |  | -3 |
| *trn-A* | F | 5,903-5,969 | 67 |  | 5,933-5,935/TGC | 4 |
| *trn-R* | F | 5,969-6,031 | 63 |  | 5,998-6,000/TCG | -1 |
| *trn-N* | F | 6,034-6,100 | 67 |  | 6,065-6,067/GTT | 2 |
| *trn-S1* | F | 6,101-6,167 | 67 |  | 6,126-6,128/GCT | 0 |
| *trn-E* | F | 6,168-6,233 | 66 |  | 6,198-6,200/TTC | 0 |
| *trn-F* | R | 6,256-6,322 | 67 |  | 6,289-6,287/GAA | 32 |
| *nad5* | R | 6,323-8,057 | 1,735 | ATT/T-- |  | 0 |
| *trn-H* | R | 8,055-8,120 | 66 |  | 8,090-8,088/GTG | -3 |
| *nad4* | R | 8,121-9,461 | 1,341 | ATG/TAA |  | 0 |
| *nad4L* | R | 9,455-9,748 | 294 | ATA/TAA |  | -7 |
| *trn-T* | F | 9,754-9,819 | 66 |  | 9,784-9,786/TGT | 5 |
| *trn-P* | R | 9,820-9,885 | 66 |  | 9,855-9,853/TGG | 0 |
| *nad6* | F | 9,888-10,412 | 525 | ATT/TAA |  | 2 |
| *cob* | F | 10,412-11,548 | 1,137 | ATG/TAG |  | -1 |
| *trn-S2* | F | 11,553-11,621 | 69 |  | 11,583-11,585/TGA | 4 |
| *nad1* | R | 11,643-12,584 | 942 | TTG/TAA |  | 21 |
| *trn-L2* | R | 12,586-12,650 | 65 |  | 12,621-12,619/CTA | 1 |
| *rrnL-16S* | R | 12,651-13,977 | 1,327 |  |  | 0 |
| *trn-V* | R | 13,978-14,049 | 72 |  | 14,016-14,014/TAC | 0 |
| *rrnS-12S* | R | 14,050-14,837 | 788 |  |  | 0 |
| *D-loop* | F | 14,838-15,648 | 811 |  |  | 0 |
