# Supplementary material for: Eighteen mitochondrial genomes of Syrphidae (Insecta: Diptera: Brachycera) with a phylogenetic analysis of Muscomorpha
Source: PLoS One. 2023 Jan 5;18(1):e0278032. doi: 10.1371/journal.pone.0278032 (PMC9815649; doi:10.1371/journal.pone.0278032)
Supplement: S18 Table — (DOCX) [file pone.0278032.s077.docx]

**Supplementary Table 18** Gene organization of the complete mitogenome of *Phytomia errans*

| Gene | Direction | Location | Size (bp) | Anticodon | Start/stop codon | Intergennic nucleotide |
| --- | --- | --- | --- | --- | --- | --- |
| *trn-l* | F | 1-66 | 66 |  | 30-32/GAT | 0 |
| *trn-Q* | R | 67-135 | 69 |  | 105-103/TTG | 0 |
| *trn-M* | F | 141-209 | 69 |  | 171-173/CAT | -5 |
| *nad2* | F | 210-1,232 | 1,023 | ATT/TAA |  | 0 |
| *trn-W* | F | 1,232-1,299 | 68 |  | 1,262-1,264/TCA | -1 |
| *trn-C* | R | 1,292-1,358 | 67 |  | 1,329-1,327/GCA | -8 |
| *trn-Y* | R | 1,372-1,438 | 67 |  | 1,407-1,405/GTA | 13 |
| *cox1* | F | 1,473-2,975 | 1,503 | ATT/TAA |  | 34 |
| *trn-L1* | F | 2,971-3,036 | 66 |  | 3,000-3,002/TAA | -5 |
| *cox2* | F | 3,040-3,723 | 684 | ATG/TAA |  | 3 |
| *trn-K* | F | 3,725-3,795 | 71 |  | 3,755-3,757/CTT | 1 |
| *trn-D* | F | 3,809-3,875 | 67 |  | 3,840-3,842/GTC | 13 |
| *atp8* | F | 3,876-4,037 | 162 | ATT/TAA |  | 0 |
| *atp6* | F | 4,034-4,708 | 675 | ATA/TAA |  | -4 |
| *cox3* | F | 4,715-5,503 | 789 | ATG/TAA |  | 6 |
| *trn-G* | F | 5,507-5,574 | 68 |  | 5,536-5,538/TCC | 3 |
| *nad3* | F | 5,575-5,928 | 354 | ATC/TAG |  | 0 |
| *trn-A* | F | 5,927-5,994 | 68 |  | 5,958-5,960/TGC | -2 |
| *trn-R* | F | 5,994-6,057 | 64 |  | 6,023-6,025/TCG | -1 |
| *trn-N* | F | 6,059-6,125 | 67 |  | 6,090-6,092/GTT | 1 |
| *trn-S* | F | 6,126-6,192 | 67 |  | 6,148-6,150/GCT | 0 |
| *trn-E* | F | 6,198-6,263 | 66 |  | 6,228-6,230/TTC | 5 |
| *trn-F* | R | 6,286-6,353 | 68 |  | 6,320-6,318/GAA | 22 |
| *nad5* | R | 6,356-8,088 | 1,733 | ATT/T-- |  | 2 |
| *trn-H* | R | 8,086-8,151 | 66 |  | 8,118-8,116/GTG | -3 |
| *nad4* | R | 8,152-9,490 | 1,339 | ATG/T-- |  | 0 |
| *nad4L* | R | 9,484-9,780 | 297 | ATG/TAA |  | -7 |
| *trn-T* | F | 9,783-9,848 | 66 |  | 9,813-9,815/TGT | 2 |
| *trn-P* | R | 9,849-9,914 | 66 |  | 9,884-9,882/TGG | 0 |
| *nad6* | F | 9,917-10,441 | 525 | ATC/TAA |  | 2 |
| *cob* | F | 10,445-11,581 | 1,137 | ATG/TAA |  | 3 |
| *trn-S2* | F | 11584-11,651 | 68 |  | 11,596-1,1598/TGA | 2 |
| *nad1* | R | 11,673-12,614 | 942 | TTG/TAA |  | 21 |
| *trn-L2* | R | 12,616-12,680 | 65 |  | 12,651-12,649/TAG | 1 |
| *rrnL-16S* | R | 12,681-14,018 | 1,338 |  |  | 0 |
| *trn-V* | R | 14,019-14,090 | 72 |  | 14,057-14,055/TAC | 0 |
| *rrnS-12S* | R | 14,091-14,878 | 788 |  |  | 0 |
| *D-loop* |  | 14,879-15,928 | 1,050 |  |  | 0 |
